# Assumptions about patients seeking PrEP: Exploring the effects of patient and sexual partner race and gender identity and the moderating role of implicit racism

**DOI:** 10.1371/journal.pone.0270861

**Published:** 2022-07-01

**Authors:** Samuel R. Bunting, Brian A. Feinstein, Sarah K. Calabrese, Aniruddha Hazra, Neeral K. Sheth, Alex F. Chen, Sarah S. Garber

**Affiliations:** 1 Department of Psychiatry and Behavioral Neuroscience, The University of Chicago Medicine, Chicago, Illinois, United States of America; 2 Department of Psychology, College of Health Professions, Rosalind Franklin University, North Chicago, Illinois, United States of America; 3 Department of Psychological and Brain Sciences, The George Washington University, Washington, District of Columbia, United States of America; 4 Section of Infectious Diseases and Global Health, Department of Medicine, The University of Chicago Medicine, Chicago, Illinois, United States of America; 5 Department of Psychiatry and Behavioral Sciences, Rush University Medical Center, Chicago, Illinois, United States of America; 6 Chicago Medical School, Rosalind Franklin University, North Chicago, Illinois, United States of America; 7 Department of Pharmaceutical Sciences, College of Pharmacy, Rosalind Franklin University, North Chicago, Illinois, United States of America; University of California San Diego, UNITED STATES

## Abstract

**Introduction:**

Daily pre-exposure prophylaxis (PrEP) for HIV-prevention is an essential component of national plans to end the HIV epidemic. Despite its well-documented safety and effectiveness, PrEP prescription has not met the public health need. Significant disparities between White and Black people exist with respect to PrEP prescription, as do disparities between men and women. One factor contributing to these disparities is clinicians’ assumptions about patients seeking PrEP.

**Methods:**

The present study sought to investigate medical students’ assumptions about patients seeking PrEP (anticipated increased condomless sex, extra-relational sex, and adherence to PrEP), and assumed HIV risk when presenting with their sexual partner. We systematically varied the race (Black or White) and gender (man or woman) of a fictional patient and their sexual partner. All were in serodifferent relationships including men who have sex with men (MSM), women (MSW), and women who have sex with men (WSM). Participants also completed an implicit association test measuring implicit racism against Black people. We evaluated the moderation effects of patient and partner race on assumptions as well as the moderated moderation effects of implicit racism.

**Results:**

A total of 1,472 students participated. For MSM patients, having a Black partner was associated with higher assumed patient non-adherence to PrEP compared to a White partner, however a White partner was associated with higher assumed HIV risk. For MSW patients, a White male patient was viewed as being more likely to engage in more extra-relational sex compared to a Black male patient. For WSM patients, White women were assumed to be more likely to have condomless and extra-relational sex, be nonadherent to PrEP, and were at higher HIV risk. Overall, implicit racism was not related to negative assumptions about Black patients as compared to White patients based on patient/partner race.

**Discussion:**

Medical education about PrEP for HIV prevention must ensure future health professionals understand the full range of patients who are at risk for HIV, as well as how implicit racial biases may affect assumptions about patients in serodifferent couples seeking PrEP for HIV prevention. As gatekeepers for PrEP prescription, clinicians’ assumptions about patients seeking PrEP represent a barrier to access. Consistent with prior research, we identified minimal effects of race and implicit racism in an experimental setting.

## Background

HIV incidence in the United States remains high, with over 36,000 diagnoses in 2019 [1]. New HIV diagnoses are made disproportionately in men who have sex with men (MSM) and people of color [1]. Daily pre-exposure prophylaxis (PrEP) with emtricitabine/tenofovir disoproxil fumarate (TDF/FTC) has the potential to significantly reduce HIV incidence, with up to 99% effectiveness with daily dosing [2–5]. Despite this well-documented effectiveness, PrEP prescription has lagged, with only 10–25% of people with HIV risk-factors prescribed PrEP [6–11].

Prescription is exceptionally inadequate for Black MSM, who accounted for approximately 25% of new HIV diagnoses in 2018, but only about 1% of PrEP prescriptions [1, 12–15] From a population standpoint, Black MSM have up to a 50% lifetime risk of acquiring HIV [16] While MSM are at increased HIV-risk relative to other populations, heterosexual people with risk-factors are also at risk for HIV and indicated for PrEP [2]. In 2018, over 6,000 HIV diagnoses were made among women who have sex with men (WSM), approximately 4,000 of whom were Black WSM, however, PrEP prescription to WSM is lagging compared to MSM [1, 2, 17, 18]. An estimated 170,000 heterosexual women in the U.S. may be PrEP candidates, but under 12,000 are prescribed PrEP [9, 19]. These numbers may underestimate the true number of WSM with PrEP indications given that guidelines may disqualify WSM seeking PrEP, even when they report recent condomless sex [20]. Previous work has also found clinicians may underestimate the HIV risk of WSM with HIV risk-factors, specifically when caring for Black women [21, 22]. Additionally, nearly 3,000 new HIV diagnoses were made in men who have sex with women (MSW) [1].

An additional avenue for increasing PrEP prescription is targeting of risk-reduction interventions to couples, particularly people in serodifferent sexual relationships [23, 24]. Recent estimates suggest that up to 70% of new HIV diagnoses made among MSM were transmitted from a primary sexual partner [25, 26]. This makes targeting services to serodifferent couples an important area for biomedical HIV prevention strategies to reduce HIV incidence and disparities [27]. Further underscoring this importance, the CDC PrEP guidelines make specific recommendation to prescribe PrEP to MSM and all people in serodifferent relationships [2]. Previous research has also shown that serodifferent relationship status may be a primary driver for patients to seek PrEP to mitigate the risk of HIV transmission to the HIV-negative partner [28]. Patients in serodifferent relationships have expressed interest in PrEP as a method for protection from HIV and to decrease anxiety about HIV transmission during sex [29].

Barriers operating at the patient-provider level, including assumptions that clinicians make about patients seeking PrEP, accompany and may exacerbate systemic factors affecting PrEP uptake, like patients’ distrust, lack of access to care, and lack of awareness of PrEP [30–32]. Assumptions of non-adherence to PrEP is one of the key reasons clinicians cite for not prescribing PrEP [33]. Assumptions that patients taking PrEP will increase their frequency of condomless sex or number of sexual partners if prescribed PrEP, sometimes termed ‘risk-compensation,’ is another clinician barrier to PrEP prescription [34–37]. Confidence in skills and time during clinic visits to adequately assess sexual activity and HIV risk are an additional clinician barrier to PrEP prescription [33]. While many of these assumptions have been studied among individual patients, it is unknown how healthcare providers’ assumptions may manifest when caring for a patient who presents with their sexual partner and whether their partner’s characteristics may influence provider assumptions [36, 38, 39].

Intersectionality is the theory that social identities confer stigma not in a simple additive way, but rather as unique experiences of discrimination and oppression for people who identify with multiple marginalized identities [40]. Social identities are thus interlinked and contribute in unique ways to the experience of stigma that patients may experience when accessing healthcare [41]. Existing along with intersectionality theory is work suggesting that the discrimination and stigma experienced by sexual and racial minority people in relationships may be different than that experienced by single people with the same identities. Specifically for same-sex couples, the minority stress and stigma that is experienced by people who are in same-sex relationships may be different than the experience of that stigma for single people [42, 43]. This is compounded for relationships interracial, same-sex couples [44]. Previous research has also identified that serodifferent HIV-status among a couple introduces a new and unique stigmatizing element for each person [45].

Understanding the factors that mitigate clinicians’ assumptions about patients seeking PrEP is essential for efforts to scale-up PrEP, as access to PrEP is at the discretion of the provider. Specifically, the role of social stigma, including implicit biases, regarding patients seeking and using PrEP is an area of active investigation, to identify specific areas where interventions are needed [46–48]. Social biases may manifest in clinical encounters as a mechanism by which clinicians may assess a patients’ presenting symptoms and requests, which is a particularly salient factor for therapies like PrEP for which prescription is at the discretion of the clinician [49].

Previous research focused on medical students (i.e., future providers) found that when a patient was assumed increase sexual risk behaviors if prescribed PrEP, then they are less likely to be willing to prescribe PrEP [50–52]. One study found that a fictional Black MSM patient was assumed to be more likely than a White MSM patient to engage in condomless sex if prescribed PrEP [50]. However, a second study did not replicate the patient race effect or identify effects of implicit racism [51]. One of the only studies to examine the role of patient gender identity in assumptions found participants also viewed the cisgender female patient as the most likely to increase frequency of condomless sex if prescribed PrEP, however the race of the patient was not specified [52]. The WSM was also viewed as being at the lowest HIV risk [52].

A recent, vignette-based study conducted with healthcare providers found that higher assumed non-adherence was associated with lower willingness to counsel patients about PrEP among providers expressing high levels of explicit racism [53]. However, this study did not find that assumptions of risk compensation were associated with willingness to counsel a patient about PrEP [53]. Another recent study conducted among healthcare providers found that greater anticipated adherence to PrEP by a fictional MSM patient served as a mechanism by which clinicians were more likely to prescribe PrEP relative to an MSW patient [54]. This study utilized a fictional patient medical record to convey relevant information [54].

These previous studies were somewhat limited in their scope and evaluation of assumptions about patients seeking PrEP. First, previous student-focused studies were limited by small, relatively homogenous samples of allopathic medical students [50, 51]. Second, vignette-based studies conducted with medical students focused largely on MSM patients, which overlooks both men and women at risk for HIV from heterosexual contact, representing a critical gap in our understanding of clinicians’ assumptions about patients seeking PrEP [50, 51]. Studies conducted among practicing healthcare providers also focused on small samples (<200) [53, 54]. While these studies did include a broader range of patients (MSM, MSW, PWID, WSM) all focused only on an individual patient rather than couples [50–54]. Given the differential experiences of stigma for interracial couples, same-sex couples, and interracial same-sex couples, investigations of how assumptions about patients seeking PrEP when presenting with their sexual partner is a notable gap in the current literature.

Specifically, the present study sought to investigate: 1) Does patient race affect medical students’ assumptions about sexual behaviors, anticipated adherence, or HIV risk, for patients seeking PrEP? 2) Does the race of a patient’s partner affect medical students’ assumptions about sexual behaviors, anticipated adherence, and HIV risk for the patient seeking PrEP? 3) Does implicit racism moderate the association between patient and/or partner characteristics and medical students’ assumptions about patients seeking PrEP?

### Hypotheses

We hypothesized: 1) Black patients will be assumed to engage in more condomless sex and extra-relational sex, and be more likely to be non-adherent to PrEP if prescribed compared to White patients; 2) Presentation with a Black partner will lead participants to assume the patient to be more likely to engage in more condomless sex and extra-relational sex, and be more likely to be non-adherent to PrEP if prescribed compared to presentation with a White partner; 3) Couples that are White-White will be assumed to be at lower HIV risk as compared to couples comprised of patients/partners of different races; 4) Implicit racial bias against Black people will be associated with negative assumptions of Black patients and partners relative to White patients and partners.

## Methods

### Participants and recruitment

Information about the study was distributed to students at 16 U.S. medical schools (10 allopathic, 6 osteopathic) with combined enrollment of 12,660 students between October 2020-February 2021. This study was conducted as a part of a larger investigation, and a total of 9,495 of these students were randomized to complete this portion of the study [55]. School administrators emailed information about the study to students with a single follow-up email reminder two weeks after the initial message. Potential participants were invited to review information about the study and were offered the option to provide their contact information if they were interested in participating. Inclusion criteria were: 1) at least 18 years of age, 2) currently enrolled in an allopathic or osteopathic medical education program in the U.S., and 3) ability to complete the study on a device with a physical keyboard (required for the Implicit Association Test [IAT]). We deliberately avoided describing the study focus on HIV and PrEP in the initial information message to reduce the risk of selection bias. Those who indicated interest and met inclusion criteria were sent a separate email message which contained a unique link to access the study. The study was hosted via Qualtrics® (Provo, UT). After completing the study, participants were given a $10.00 credit for an online retailer and sent a debrief message.

### Study procedures

Examples of all study materials are included as S1 File. Prior to widespread survey distribution, a focus group of 10 allopathic medical students reviewed and provided feedback on the patient vignettes and study measures. Minor edits to item wording and study platform mechanics were made based on focus group comments.

**Fig 1:**
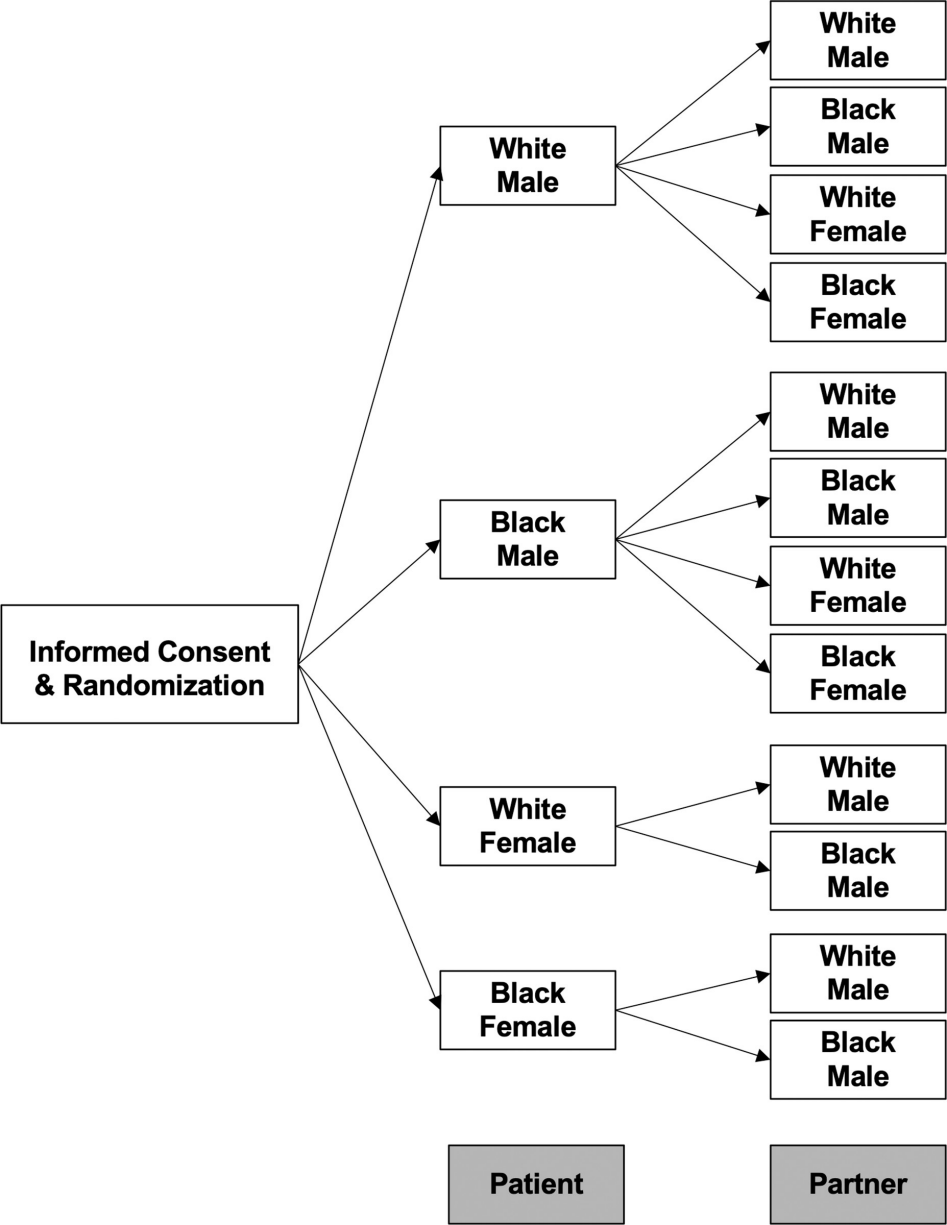
Organization of the study including the randomization process and the organization of the 12 individual study conditions. Following completion of the informed consent, participants were randomized to one of the patient couple presentations, which were comprised of the combinations depicted.

Following completion of the informed consent and eligibility criteria items, participants were randomized to one of 12 experimental conditions shown in Fig 1. These conditions represented various combinations of patients and partners of differing races and genders, representing three key populations for PrEP prescription (MSM, MSW, WSM). Randomization was completed using the Qualtrics® randomization algorithm programmed to ensure balanced numbers of participants in each of the 12 experimental conditions. Following randomization, participants were presented with a vignette and accompanying clinical information.

#### Patient vignette

**Fig 2:**
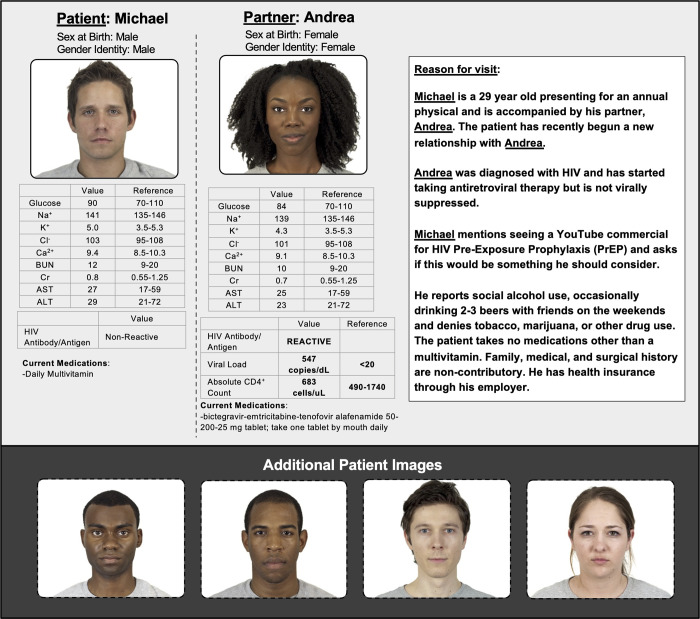
Example vignette presentations are shown. All patients were either presented as Michael if male or Michelle if female and their partner was presented as either Andrew if male or Andrea if female. The underlining in the figure is for emphasis only and was not included in the experiment. All patients and their partners were presented as cisgender, as indicated by matching sex at birth and gender identity. Additional patient images are shown in the bottom pane of the figure, which were used when there were MSM couples of either two Black MSM or two White MSM, or when the patient was presented with a White female partner. All remaining details were identical across vignette presentations.

The patient vignettes serve as the key method for systematic variation of the experimentally relevant variables. The vignette and accompanying information presented a fictional patient and their partner (Fig 2), with systematic variation of patient race (Black vs. White), patient gender identity (cisgender man vs. cisgender woman, inferred by reported sex at birth and gender identity both being specified as “male” or both being specified as “female”), partner race (Black vs. White), and partner gender identity (cisgender man vs. cisgender woman, inferred the same way as patient gender identity). Patient and partner combinations represented MSM, MSW, and WSM. Women who have sex with women were not represented given their low risk for HIV [56]. An image was presented for both the patient and the partner. Images were taken from the Chicago Faces Database, a publicly available resource containing standardized images of individuals across multiple genders, races, and ages [57]. The Database provides data from independent raters who provided ratings of the age and race of the people presented in the images as well as measurements of facial dimensions [57]. We matched images across experimental conditions based on facial measurements and age ratings to mitigate potential confounding effects.

Consistent with prior vignette studies, the partner was described as being HIV-positive and not being virally suppressed [51, 53, 54]. In the current study, this was indicated by laboratory data showing a detectable HIV viral load, combination antiretroviral therapy in the partner’s medication list, and explicit mention in the vignette text. HIV antigen/antibody testing results were also provided for the patient to show HIV-negative status, along with laboratory results confirming adequate kidney function to indicate PrEP would be safely tolerated. The couple was described as monogamous and sexually active with intermittent condom use, and both the patient and partner were in good overall health without comorbidities. The patient was explicitly requesting PrEP from the clinician. All vignettes were identical except for the image of the patient and their partner as well as the necessary modifications to specify gender identity (pronouns, name).

#### Vignette follow-up items (assumptions)

After reviewing the vignette and clinical information, participants were directed to complete a series of four follow-up items that reflected judgments or assumptions about the patient. Three items asked about anticipated patient behaviors if prescribed PrEP, including: “If prescribed PrEP how likely would this patient be to have *more* condomless sex?” “If prescribed PrEP how likely would this patient be to have extra-relational sex (ie. sex outside of their current relationship)?” and “If prescribed PrEP, how likely is it that this patient would adhere to the medication?” All follow-up items were rated on a 7-point scale (1 = extremely unlikely, 7 = extremely likely). Anticipated adherence was reverse scored (7 = 1) such that a higher score indicated greater assumed *non-*adherence to PrEP. The final follow-up item asked participants: “How high is this patient’s overall HIV risk without PrEP?” and was also rated on a 7-point scale (1 = extremely low, 7 = extremely high).

#### Implicit racism

Participants completed an IAT, which is a validated, computer-based tasks for measuring implicit biases, to detect implicit racism [58]. The IAT in this study were taken from the Harvard University Project Implicit publicly available IAT library and completed within the Qualtrics® study platform [59, 60]. The interpretable measurement of the IAT is the *d*-score, which ranges from -2 to +2, with positive *d*-scores indicating implicit preference for the majority group (White) and negative *d*-scores indicating implicit preference for the minority group (Black) [61]. A *d*-score of ‘0’ indicates no implicit preference for either group and higher *d*-scores are interpreted as greater implicit bias against the minority group [61].

The IAT presented participants with stimuli (images or words) on a computer screen. Participants made judgements by pairing stimuli corresponding to one group or another with attributes (positive or negative) as instructed and their reaction times were measured by the computer with a quicker reaction occurring with judgements of associated concepts. We used images of Black and White male and female faces drawn from a standard database as well as the standard verbal stimuli of positive words (eg. “pleasure,” “glorious,” or “excellent”) and negative words (eg. “failure,” “sickening,” or “sadness”).

#### Demographics

The final study section captured demographic information about participants. We inquired about sexual orientation, gender identity, race/ethnicity, year in training, academic program, and the state in which they were training. We also included a manipulation check item, which asked participants: “What was the race of the patient presented at the beginning of this study?” Participants selected a race from a multiple-choice list. This item was purposely separated from the vignette by several blocks of unrelated items. Participants ‘passed’ the manipulation check if they correctly identified the race of the patient in the condition to which they were randomized.

### Statistical analyses

Descriptive statistics were calculated to describe the variables. Pearson’s correlation coefficients were calculated between assumptions, HIV risk, and implicit racism. First, we performed a repeated measures GLM to compare the means of the three assumptions to each other. Next, we performed a series of four GLMs for each of the couple groups (MSM, MSW, WSM), one for each of the assumption items. Patient and partner race were entered as independent variables in the GLMs to evaluate the main and interaction effects of each. All GLMs controlled for respondents’ year in training, sexual orientation, gender identity, and race due to their conceptual relevance. Bonferroni post-hoc pairwise comparisons of the adjusted means were conducted to identify differences between patient and partner race as well as the interaction terms.

**Fig 3:**
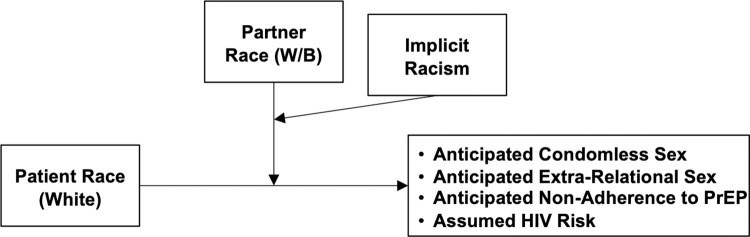
Conceptual diagrams of the moderation models. This series of moderation analyses was performed for each couple group (MSM, MSW, WSM). All analyses controlled for the gender identity of the patient and their partner, as well as participants’ year of training, sexual orientation, gender identity, and race.

Moderation analyses were conducted to evaluate the potential moderating effect of implicit racism on the effects of patient and/or partner race on assumptions about behaviors if prescribed PrEP. (Fig 3) Moderation models were analyzed utilizing Hayes’s PROCESS macro for SPSS (v3.5), using values of the moderators at the 16^th^, 50^th^, and 84^th^ percentiles [62, 63]. For all moderation analyses, we used PROCESS to calculate 10,000 bootstrapped samples to generate bias-corrected 95% confidence intervals (CIs) to evaluate the direct effects between independent and dependent variables. Moderation models also controlled for the relevant participant demographic variables. Analyses were restricted to participants who passed the manipulation check. Analyses were repeated with the full sample of participants regardless of manipulation check passage as the intrinsic nature of our race manipulation (image of patient and partner) may have precipitated differential judgment even without conscious awareness of the manipulation [64]. This replication of analysis was done to determine whether there were differences in assumptions given that previous studies have identified meaningful differences in outcomes between these participant groups [54].

All statistics were completed utilizing IBM SPSS v27 (Armonk, NY). This study was reviewed and approved by the Institutional Review Board of Rosalind Franklin University (Protocol# COP-20-256). Online informed consent to participate was obtained from all study participants.

## Results

A total of 2,973 of the 9,495 students invited indicated interest in participating in the study (response rate = 31.3%). Of these, 1,108 were prevented from beginning the study due to failure to meet one or more of the inclusion criteria, and 1,865 completed the study. We removed 222 responses due to IAT completion errors, including completion times greater than 3 standard deviations from the mean and incomplete responses. We removed 171 responses due to failure of the manipulation check leaving a final analytic sample of 1,472. The number of participants randomized to each of the 12 experimental conditions ranged from 118–125. Mean study completion time was 16.9 (*SD* = 4.3) minutes.

### Demographics

Slightly over half of participants were in allopathic medical education programs (*n* = 788, 53.5%), and the greatest proportion were in their first year of training (*n* = 429, 29.1%). Regarding race, most of the sample identified as White (*n* = 803, 54.6%), and most identified as heterosexual (*n* = 1,280, 87.0%), and as cisgender women (*n* = 850, 57.7%). Mean participant age was 25.6 (*SD* = 2.8) years. The greatest percentage of respondents were in training in the Midwestern U.S. (*n* = 720, 48.9%). Full demographic information is provided in Table 1. The overall mean of the racism IAT was 0.28 (*SD* = 0.44).

**Table 1:** Sample demographics.

	*N*	%
Sample Number	1,472	100
**Academic Program**	***n***	**%**
Allopathic Medicine (MD)	788	53.5%
Osteopathic Medicine (DO)	684	46.5%
**Year of Training**	*** ***	** **
1st	429	29.1%
2nd	424	28.8%
3rd	306	20.8%
4th+	313	21.3%
**Race/Ethnicity**		
White	803	54.6%
Black	50	3.4%
Hispanic/Latino	61	4.1%
Asian	452	30.7%
Other	106	7.2%
**Sexual Orientation**		
Heterosexual (straight)	1,280	87.0%
Homosexual (gay/lesbian)	69	4.7%
Bisexual	101	6.9%
Other	22	1.5%
**Gender Identity**		
Man (cisgender male)	604	41.0%
Woman (cisgender female)	850	57.7%
Other	18	1.2%
**Region**		
South	103	7.0%
Northeast	317	21.5%
West	332	22.6%
Midwest	720	48.9%

Demographic compositions of the 12 individual experimental conditions were also compared; no meaningful differences were identified, suggesting successful randomization. (S1 Table) Demographics were also compared between the group which passed the manipulation check and the group that failed. We found that a greater percentage of participants who passed the manipulation check identified as a sexual orientation other than heterosexual. (S2 Table).

### Assumptions

The four assumptions were moderately correlated with each other. Anticipated condomless sex and extra-relational sex were correlated with each other (*r* = 0.36, *p* < .001), as were anticipated non-adherence and anticipated extra-relational sex (*r* = 0.13, *p* < .001). Finally, assumed HIV risk was correlated with anticipated condomless sex (*r* = 0.11, *p* < .001), extra-relational sex (*r* = 0.09, *p* < .001), and non-adherence to PrEP (*r* = -0.11, *p* < .001). Implicit racism was only correlated with anticipated condomless sex (*r* = 0.05, *p* = .04).

Across all couple types, participants assumed that the patient would increase frequency of condomless sex (*M* = 4.72, 95%CI[4.66–4.79]) compared to extra-relational sex (*M* = 3.82, [3.77–3.88], *p* < .001), and non-adherence to PrEP (*M* = 2.23, [2.18–2.28], *p* < .001). Assumed engagement in extra-relational sex was greater than non-adherence to PrEP (*p* < .001).

### MSM couple conditions

#### Assumptions

In the MSM couple conditions, we did not identify any significant effect of patient race on participant assumptions, but a significant effect of partner race on two of four assumptions was identified. Specifically, having a Black partner was associated with higher assumed patient non-adherence to PrEP compared to a White partner. (Table 2) We also found that participants viewed male patients with a White male partner as being at a higher HIV risk compared to male patients with a Black male partner. (Table 2) Subsequent analysis of the interaction effects between patient and partner race identified a significant interaction with respect to one of the four assumptions: anticipated non-adherence to PrEP. Specifically, a MSM couple with a White patient and Black partner was viewed as more likely to be non-adherent to PrEP compared to a Black patient with a White partner (*p* = .04). There were no significant patient race × partner race interaction effects relative to assumptions of condomless sex, extra-relational sex, or HIV risk for the MSM couples. (Table 2)

**Table 2:** Main effects of patient and partner race on assumptions.

		Anticipated Condomless Sex		Anticipated Extra-Relational Sex		Anticipated Non-Adherence		Assumed HIV Risk	
**MSM**									
		*M* (95%CI)	*p*	*M* (95%CI)	*p*	*M* (95%CI)	*p*	*M* (95%CI)	*p*
	OVERALL	4.73 (4.63, 4.83)	-	3.82 (3.72, 3.92)	-	2.22 (2.13, 2.31)	-	5.59 (5.48, 5.70)	-
Patient Race	White	4.74 (4.59, 4.88)	*Ref*.	3.85 (3.71, 3.99)	*Ref*.	2.24 (2.12, 2.37)	*Ref*.	5.58 (5.43, 5.73)	*Ref*.
	Black	4.72 (4.57, 4.86)	.85	3.79 (3.65, 3.93)	.51	2.19 (2.06, 2.32)	.56	5.60 (5.45, 5.75)	.82
Partner Race	White	4.72 (4.57, 4.86)	*Ref*.	3.74 (3.60, 3.88)	*Ref*.	2.11 (1.99, 2.24)	*Ref*.	5.71 (5.56, 5.86)	*Ref*.
	Black	4.73 (4.59, 4.88)	.89	3.90 (3.76, 4.04)	.12	2.32 (2.19, 2.45)	**.02**	5.47 (5.32, 5.62)	**.03**
Pt. Race X Partner Race	White X White	4.68 (4.48, 4.89)	*Ref*.	3.80 (3.61, 4.00)	*Ref*.	2.15 (1.97, 2.33)	*Ref*.	5.72 (5.51, 5.93)	*Ref*.
	White X Black	4.79 (4.58, 5.00)^a^	.60	3.90 (3.70, 4.10)	.13	2.34 (2.16, 2.52)	.08	5.43 (5.22, 5.65)	.20
	Black X White	4.76 (4.55, 4.96)^a^	.44	3.68 (3.48, 3.88)	.96	2.08 (1.90, 2.26)	.79	5.70 (5.49, 5.92)	.67
	Black X Black	4.68 (4.47, 4.88)	.97	3.89 (3.70, 4.09)	.52	2.30 (2.12, 2.48)	.23	5.50 (5.29, 5.72)	.16
**MSW**									
		*M* (95%CI)	*p*	*M* (95%CI)	*p*	*M* (95%CI)	*p*	*M* (95%CI)	*p*
	OVERALL	4.77 (4.66, 4.88)	-	3.81 (3.71, 3.91)	-	2.26 (2.17, 2.35)	-	5.53 (5.42, 5.64)	-
Patient Race	White	4.82 (4.66, 4.97)	*Ref*.	3.93 (3.79, 4.07)	*Ref*.	2.34 (2.22, 2.47)	*Ref*.	5.44 (5.29, 5.59)	*Ref*.
	Black	4.71 (4.56, 4.87)	.36	3.69 (3.55, 3.83)	**.02**	2.17 (2.05, 2.30)	.06	5.61 (5.46, 5.76)	.12
Partner Race	White	4.74 (4.58, 4.90)	*Ref*.	3.84 (3.71, 3.98)	*Ref*.	2.33 (2.20, 2.46)	*Ref*.	5.50 (5.35, 5.65)	*Ref*.
	Black	4.79 (4.63, 4.94)	.67	3.78 (3.64, 3.91)	.50	2.19 (2.06, 2.31)	.12	5.56 (5.41, 5.71)	.60
Pt. Race X Partner Race	White X White	4.77 (4.55, 4.99)	*Ref*.	3.95 (3.76, 4.15)	*Ref*.	2.46 (2.28, 2.63)	*Ref*.	5.35 (5.14, 5.56)	*Ref*.
	White X Black	4.86 (4.64, 5.08)	.94	3.90 (3.71, 4.10)	.55	2.23 (2.05, 2.41)	.63	5.54 (5.33, 5.75)	.64
	Black X White	4.71 (4.48, 4.93)	.38	3.73 (3.54, 3.93)	.07	2.20 (2.02, 2.39)	.49	5.65 (5.43, 5.87)	.79
	Black X Black	4.72 (4.50, 4.94)	.73	3.65 (3.45, 3.84)	**.03**	2.14 (1.96, 2.32)	**.02**	5.58 (5.36, 5.79)	.14
**WSM**									
		*M* (95%CI)	*p*	*M* (95%CI)	*p*	*M* (95%CI)	*p*	*M* (95%CI)	*p*
	OVERALL	4.67 (4.55, 4.79)	-	3.83 (3.73, 3.93)	-	2.20 (2.11, 2.29)	-	5.49 (5.38, 5.60)	-
Patient Race	White	4.82 (4.66, 4.98)	*Ref*.	3.98 (3.84, 4.13)	*Ref*.	2.31 (2.18, 2.43)	*Ref*.	5.61 (5.46, 5.77)	*Ref*.
	Black	4.53 (4.36, 4.69)	**.01**	3.68 (3.54, 3.82)	**.003**	2.09 (1.97, 2.22)	**.02**	5.38 (5.23, 5.53)	**.03**
Partner Race	White	4.68 (4.51, 4.84)	*Ref*.	3.80 (3.66, 3.95)	*Ref*.	2.24 (2.12, 2.36)	*Ref*.	5.52 (5.37, 5.67)	*Ref*.
	Black	4.67 (4.51, 4.83)	.96	3.86 (3.72, 4.00)	.58	2.16 (2.03, 2.28)	.34	5.47 (5.32, 5.62)	.67
Pt. Race X Partner Race	White X White	4.76 (4.53, 4.99)	*Ref*.	4.02 (3.82, 4.22)^b2^	*Ref*.	2.36 (2.19, 2.54)	*Ref*.	5.68 (5.47, 5.90)	*Ref*.
	White X Black	4.88 (4.65, 5.11)^b^	.40	3.95 (3.75, 4.15)^b1^	.19	2.25 (2.07, 2.42)	.65	5.55 (5.34, 5.76)^a^	.80
	Black X White	4.60 (4.37, 4.83)	**.01**	3.59 (3.39, 3.79)^b1,b2^	.24	2.12 (1.94, 2.29)	.14	5.36 (5.14, 5.57)^a^	.33
	Black X Black	4.46 (4.23, 4.69)^b^	.07	3.78 (3.58, 3.98)	.09	2.06 (1.89, 2.24)	**.02**	5.40 (5.18, 5.61)	.07

This represents the adjusted means considering only the main effects of patient race and partner race. Separate models were constructed for each couple type (MSM, MSW, or WSM) and outcome (12 models in total). Models were adjusted for the following variables: respondents’ year in training, sexual orientation, gender identity, and race. MSM = men who have sex with men; WSM = women who have sex with men; MSW = men who have sex with women.

a = difference is significant at *p* < .05

b = difference is significant at *p* < .01.

#### Moderating effect of implicit racism

In moderation analyses (Fig 3 & Table 3) we found that implicit racism moderated the effect between patient and partner race on anticipated condomless sex. Specifically, we found that when a Black patient was presented with a White male partner the patient was viewed as more likely to have condomless sex by participants expressing high levels of implicit racism (*b* = 0.53, *p* = .01) compared to a White patient with a White male partner. No additional moderating effects of implicit racism were identified on assumptions.

**Table 3:** Moderation analyses.

	Anticipated Condomless Sex				Anticipated Extra-Relational Sex			
	Partial Effect (95%CI)	*p*	Conditional Effect (95%CI)	*p*	Partial Effect (95%CI)	*p*	Conditional Effect (95%CI)	*p*
**MSM**								
Patient Race (White)	0.02 (-0.19, 0.22)	.88	-0.05 (-0.83, 0.74)	.91	0.06 (-0.15, 0.26)	.59	0.39 (-0.37, 1.15)	.31
Partner Race	-0.02 (-0.23, 0.19)	.86	0.05 (-0.29, 0.38)	.79	-0.17 (-0.37, 0.04)	.11	0.01 (-0.31, 0.34)	.93
Patient Race X Partner Race	-	-	-0.05 (-0.54, 0.43)	.82	-	-	-0.26 (-0.72, 0.21)	.28
Racism	0.00 (-0.23, 0.23)	.99	0.82 (-0.21, 1.85)	.12	-0.02 (-0.24, 0.21)	.89	0.78 (-0.22, 1.77)	.13
Patient Race X Racism	-	-	-1.06 (-2.54, 0.41)	.16	-	-	-0.96 (-2.38, 0.47)	.19
Partner Race X Racism	-	-	-0.65 (-1.29, -0.02)	**.04**	-	-	-0.39 (-1.00, 0.23)	.22
Patient Race X Partner Race X Racism	-	-	1.01 (0.09, 1.94)	**.03**	-	-	0.47 (-0.42, 1.36)	.30
**MSW**								
Patient Race (White)	-0.12 (-0.34, 0.11)	.31	0.16 (-0.69, 1.02)	.71	-0.24 (-0.44, -0.04)	**.02**	0.15 (-0.61, 0.91)	.70
Partner Race	-0.04 (-0.26, 0.18)	.73	-0.01 (-0.88, 0.86)	.99	0.07 (-0.13, 0.27)	.48	0.04 (-0.73, 0.82)	.92
Patient Race X Partner Race	-	-	-0.05 (-0.59, 0.49)	.86	-	-	0.03 (-0.45, 0.51)	.90
Racism	0.31 (0.05, 0.57)	**.02**	-0.24 (-3.03, 2.54)	.86	0.02 (-0.21, 0.26)	.85	-0.76 (-3.24, 1.71)	.54
Patient Race X Racism	-	-	0.25 (-1.46, 1.95)	.78	-	-	0.61 (-0.91, 2.13)	.43
Partner Race X Racism	-	-	0.29 (-1.44, 2.02)	.74	-	-	0.37 (-1.17, 1.90)	.64
Patient Race X Partner Race X Racism	-	-	-0.12 (-1.18, 0.95)	.83	-	-	-0.31 (-1.25, 0.64)	.53
**WSM**								
Patient Race (White)	0.31 (0.08, 0.54)	**.009**	-0.33 (-1.24, 0.58)	.47	0.32 (0.12, 0.53)	**.002**	-0.04 (-0.83, 0.75)	.92
Partner Race	0.01 (-0.22, 0.25)	.90	0.04 (-0.35, 0.42)	.85	-0.05 (-0.25, 0.15)	.64	0.08 (-0.25, 0.41)	.64
Patient Race X Partner Race	-	-	0.05 (-0.52, 0.62)	.86	-	-	-0.21 (-0.70, 0.29)	.41
Racism	0.218 (-0.05, 0.49)	.11	1.23 (0.05, 2.40)	**.04**	0.16 (-0.08, 0.39)	.18	0.12 (-0.90, 1.15)	.82
Patient Race X Racism	-	-	-1.36 (-3.09, 0.36)	.12	-	-	0.32 (-1.18, 1.83)	.67
Partner Race X Racism	-	-	-0.63 (-1.38, 0.13)	.11	-	-	-0.01 (-0.67, 0.65)	.98
Patient Race X Partner Race X Racism	-	-	0.80 (-0.28, 1.88)	.15	-	-	-0.14 (-1.09, 0.80)	.76
	**Anticipated Non-Adherence**				**Assumed HIV Risk**			
	Partial Effect (95%CI)	*p*	Conditional Effect (95%CI)	*p*	Partial Effect (95%CI)	*p*	Conditional Effect (95%CI)	*p*
**MSM**								
Patient Race (White)	-0.03 (-0.21, 0.15)	.73	0.12 (-0.56, 0.81)	.72	-0.01 (-0.22, 0.21)	.94	0.24 (-0.58, 1.06)	.57
Partner Race	0.20 (0.01, 0.38)	**.04**	0.19 (-0.11, 0.48)	.22	0.25 (0.03, 0.46)	**.03**	0.25 (-0.10, 0.60)	.17
Patient Race X Partner Race	-	-	-0.08 (-0.50, 0.35)	.73	-	-	-0.15 (-0.66, 0.36)	.57
Racism	-0.04 (-0.24, 0.16)	.71	-0.07 (-0.98, 0.84)	.88	0.17 (-0.07, 0.40)	.18	-0.16 (-1.25, 0.92)	.77
Patient Race X Racism	-	-	-0.46 (-1.76, 0.83)	.48	-	-	-0.19 (-1.73, 1.36)	.81
Partner Race X Racism	-	-	-0.04 (-0.60, 0.51)	.87	-	-	0.22 (-0.45, 0.89)	.52
Patient Race X Partner Race X Racism	-	-	0.44 (-0.37, 1.25)	.29	-	-	0.15 (-0.81, 1.12)	.76
**MSW**								
Patient Race (White)	-0.17 (-0.35, 0.01)	.07	0.17 (-0.52, 0.86)	.63	0.17 (-0.05, 0.38)	.13	0.26 (-0.56, 1.09)	.53
Partner Race	0.15 (-0.03, 0.33)	.11	-0.06 (-0.76, 0.65)	.87	-0.06 (-0.27, 0.16)	.62	0.25 (-0.59, 1.09)	.56
Patient Race X Partner Race	-	-	-0.13 (-0.56, 0.30)	.56	-	-	-0.17 (-0.69, 0.35)	.52
Racism	-0.05 (-0.27, 0.17)	.64	-0.05 (-2.31, 2.20)	.96	0.07 (-0.18, 0.33)	.57	0.37 (-2.32, 3.07)	.78
Patient Race X Racism	-	-	-0.34 (-1.73, 1.04)	.62	-	-	-0.02 (-1.67, 1.64)	.99
Partner Race X Racism	-	-	0.61 (-0.79, 2.01)	.39	-	-	0.46 (-1.21, 2.13)	.59
Patient Race X Partner Race X Racism	-	-	-0.11 (-0.98, 0.75)	.79	-	-	-0.42 (-1.45, 0.61)	.42
**WSM**								
Patient Race (White)	-0.23 (-0.41, -0.06)	**.01**	0.69 (0.00, 1.38)	**.05**	0.24 (0.03, 0.46)	**.03**	-0.24 (-1.09, 0.60)	.57
Partner Race	-0.08 (-0.25, 0.10)	.40	0.02 (-0.27, 0.31)	.91	0.06 (-0.16, 0.27)	.60	0.15 (-0.21, 0.50)	.42
Patient Race X Partner Race	-	-	-0.26 (-0.69, 0.17)	.24	-	-	-0.05 (-0.58, 0.48)	.84
Racism	-0.19 (-0.39, 0.02)	.07	0.63 (-0.26, 1.52)	.16	0.00 (-0.25, 0.25)	.98	-0.03 (-1.12, 1.06)	.96
Patient Race X Racism	-	-	-1.79 (-3.10, -0.48)	**.01**	-	-	0.73 (-0.87, 2.34)	.37
Partner Race X Racism	-	-	-0.49 (-1.07, 0.08)	.09	-	-	-0.06 (-0.76, 0.65)	.87
Patient Race X Partner Race X Racism	-	-	1.05 (0.23, 1.87)	**.01**	-	-	-0.31 (-1.31, 0.70)	.55

The coefficients for the moderation analyses. A series of four moderation models were conducted (Fig 3) for each couple type (MSM, MSW, WSM) to evaluate the effects of patient and partner race as well as potential moderating effects of implicit racism on assumptions and assumed HIV risk. For all moderation analyses, White patient race was taken as the reference group. Racism indicates the implicit racism IAT *d*-score. All moderation models controlled for respondents’ year in training, sexual orientation, gender identity, and race. MSM = men who have sex with men; WSM = women who have sex with men; MSW = men who have sex with women.

### MSW couple conditions

#### Assumptions

In the MSW couple conditions, we identified a single effect of patient race on assumed extra-relational sex in the setting of PrEP prescription, specifically finding that a White male patient was assumed to engage in more extra-relational sex compared to a Black male patient if prescribed PrEP (*p* = .02). (Table 2) No effects of partner race were identified on any of the four assumptions. Analysis of the interaction effects of patient and partner race revealed that a White male patient with a White female partner was viewed as more likely to engage in extra-relational sex and more likely to exhibit non-adherence to PrEP compared to a Black male patient with a Black female partner. (Table 2)

#### Moderating effect of implicit racism

No moderating effects of implicit racism were identified on the relationship between patient race, partner race, or the interaction between patient and partner race on any of the four assumptions for the MSW conditions. (Table 3)

### WSM couple conditions

#### Assumptions

In the WSM conditions, we found that a White female patient was assumed to engage in more condomless sex, extra-relational sex, non-adherence to PrEP, and was assumed to be at higher HIV risk as compared to a Black female patient. (Table 2) No effects of partner race were identified on any of the four assumptions. Evaluating the interaction between patient and partner race revealed that a White female patient with a Black partner was viewed as more likely to engage in condomless sex compared to a Black female patient with a Black partner. A White female patient with a White partner was viewed as more likely to engage in extra-relational sex as compared to a Black female patient with a White partner. A White female patient with a Black partner was viewed as more likely to have extra-relational sex and at higher HIV risk. Regarding assumed non-adherence to PrEP, we found that a White female patient with a White partner was viewed as more likely to be non-adherent to PrEP compared to a Black female with a Black partner.

#### Moderating effect of implicit racism

We identified a single, moderating effect of implicit racism on the interaction between patient race and partner race. (Table 3) Specifically, when a Black Woman was presented with a White male partner she was viewed as being more likely to adhere to PrEP (less likely to exhibit non-adherence) at moderate (*b* = -0.27, *p* = .04) and high levels (*b* = -0.40, *p* = .03) of implicit racism compared to a White female patient with a White male partner.

### Replication of analyses with full sample

We performed an additional set of analyses to compare the four assumptions and implicit racism between the groups of participants who passed the manipulation check and the group that failed. (S2 File) The mean of the implicit racism measure did not differ between those who passed (*M* = 0.28, *SD* = 0.44) or failed the manipulation check (*M* = 0.34, *SD* = 0.41, *p* = .11). We also found significant differences in the means of several of the assumption items when comparing between the groups of participants. Specifically, we found that assumed extra-relational sex was higher among those who failed the manipulation check (*M* = 4.07, [3.89–4.25]) compared to those who passed (*M* = 3.82, [3.76–3.88], *p* = .007) as was assumed non-adherence to PrEP (*M* = 2.60, [2.42–2.78] vs. *M* = 2.22, [2.17–2.28], *p* < .001). Assumed condomless sex was higher among those who failed the manipulation check (*M* = 4.91, [4.74–5.08] vs. *M* = 4.72, [4.66–4.78]) but was not statistically significant (*p* = .06). Assumed HIV risk also did not differ significantly between the two groups, however, was slightly higher among those who passed the manipulation check (*M* = 5.36, [5.17–5.55] vs. *M* = 5.54, [5.48–5.60], *p* = .07).

All study analyses were repeated all analyses with the full sample (*n* = 1,643), including those who did not pass the race manipulation check item. (S2 File) Overall, the pattern of findings was largely the same between the restricted and full analytic samples. We did find that including participants who failed the manipulation check led to the assumption that a Black male with a Black female partner would be more likely to not adhere to PrEP if prescribed (*M* = 2.45 [2.28–2.63]) compared to a White male with a White female partner (*M* = 2.19 [2.01–2.36], *p* = .04). There were no additional findings regarding the moderating role of implicit racism when repeating the analyses with the full analytic sample. (S2 File).

## Discussion

Preventing HIV with PrEP is an essential component of the American *Ending the HIV Epidemic* plan [65]. Achieving the goals of this plan requires addressing barriers to prescribing PrEP to all patients with HIV risk-factors. The present study advances understanding of the role of implicit racism on assumptions about patients seeking PrEP by investigating the intersecting roles of the race of a patient and their partner.

This research represents a critical extension of prior studies of the role of implicit biases in the setting of PrEP prescription and assumptions about patients seeking PrEP. Specifically, the current study was conducted with a large, national sample of allopathic and osteopathic medical students. Second, our study extends beyond MSM patients to include WSM and MSW as patients presented with hypothetical sexual partners. We present analyses of three types of couples representing key patients at risk for HIV (MSM, MSW, WSM) using systematic variation of fictional patient and partner gender identity (eg. man/woman) within a vignette. This is an important distinction as efforts are needed to specifically address barriers to PrEP access that are unique to specific patient groups, specifically heterosexual men and women, racial minorities, and MSM [6, 66, 67]. We systematically varied the race (Black or White) of the patient and the patient’s partner in all conditions and stratify analysis by patient/partner gender groups (MSM, MSW, WSM).

Additionally, previous studies have presented patient race and gender via vignette or medical record text (eg. ‘The patient is a 31-year-old Black male’) [50–54]. We presented these demographic characteristics by presenting fictional patients via images with accompanying clinical data, which makes our design more representative of real-life clinical scenarios in which the clinician would encounter the patient in the process of evaluating and interviewing them. Finally, this study is a critical extension of previous work presenting patient information via a fictional medical record [54]. We found that systematically varying patient/partner race and patient/partner gender identity led to differential assumptions about condomless sex, extra-relational sex, anticipated non-adherence to PrEP, and assumed HIV risk, though overall there were more similarities than differences across conditions.

Across the categories of couples (MSM, MSW, WSM), we found that White patients, regardless of their gender identity and the race/gender identity of their fictional partner, were largely assumed to be more likely to engage in condomless sex, extra-relational sex, and be non-adherent to PrEP if prescribed. This was directly contrary to our hypotheses regarding patient race. The trend was particularly pronounced for White women compared to Black WSM: We found that White women were viewed as more likely to have condomless sex and extra-relational sex, and less likely to adhere to PrEP as compared to Black women.

Our pattern of findings is particularly interesting when considering previous work elucidating negative stereotypes of Black women as sexually promiscuous and irresponsible [68, 69]. The findings of the present study are in contrast to previous work with healthcare providers, which found that Black women were viewed as *more* likely not to adhere PrEP [53]. Additionally, the previous study found that an *explicit* measure of racism was associated with biased assumptions of non-adherence to PrEP among Black women [53]. We found that when a Black woman was presented with a White male partner, she was viewed as more likely to adhere to PrEP as compared to presentation with a Black male partner. When a Black woman had a White male partner, she was believed to be more likely to adhere to PrEP by participants with high levels of internalized prejudice against Black people. The differences between the current and previous studies may be explained by a difference in study population given that our work was conducted with physicians in training as compared to practitioners. This may be attributed to the recent focus on implicit bias training in medical education and that our study population was more likely to receive this kind of training during medical education and be more proximal to medical education as compared to currently practicing physicians [70–72].

In our analyses of the effects of implicit racism in the MSM patient conditions, we found that implicit racism moderated only one assumption. First, we found that a Black patient with a White male partner was viewed as more likely to have condomless sex as compared to a Black MSM with a Black male partner among participants expressing high levels of implicit racism. This finding contrasts with previous work exploring the sexual stereotypes attributed to Black men, specifically Black MSM as engaging in risky sexual activity and being irresponsible [73]. Whereas we hypothesized that participants higher in implicit bias would make more favorable assumptions about White patients compared to Black patients, they instead made more favorable assumptions about the Black patient. Other experimental studies have found minimal evidence for implicit racism affecting PrEP clinical decision-making for MSM patients [51, 54].

Overall, our findings regarding race are largely in contrast with previous studies with medical students portraying fictional MSM patients. Previous work found that medical students viewed a Black MSM patient to be more likely to have condomless sex and less likely to adhere if prescribed PrEP [50, 51]. The present study found no differences in assumed changes in behavior based solely on patient race for MSM patients. Regarding partner race, we found that having a Black male partner would lead the patient to be less adherent to PrEP, however having a White male partner was viewed as placing the patient at higher HIV risk compared to a Black partner.

For the MSW patients, patient race yielded one significant difference in anticipated behavior changes in the setting of PrEP prescription: White patient race was associated with higher assumed engagement in extra-relational sex. Regarding WSM, we found that this trend continued regarding race in that White women were assumed as the most likely to change their sexual behaviors if prescribed PrEP. Taken together, these findings suggest that participants judged White patients as more likely to change sexual behaviors in the setting of PrEP prescription. This was a unique finding given the extensive previous research describing stigmatized stereotypes of Black people, specifically regarding sexuality (eg. hypersexuality, irresponsibility) [68, 69, 73]. However, it is also important to note that previous experimental studies investigating the role of patient race have identified tenuous effects as well [50, 51, 53, 54].

### HIV risk assumptions

Throughout the results of the present study, we identified an interesting collection of findings with respect to the assumed HIV risk of the presented patient. There were instances in which White patients in a couple were viewed as more likely to engage in risk behaviors, including condomless sex, extra-relational sex, and non-adherence to PrEP, and were simultaneously assumed to be at lower HIV risk. For example, White women were viewed as being at lower HIV-risk, less likely to adhere to PrEP, and more likely to engage in extra-relational sex if prescribed PrEP. Thus, participants may have viewed White patients as being at less HIV risk and therefore comfortable engaging in activities that would increase HIV risk.

Importantly, our vignette presented the same HIV risk for all couples regardless of the race and gender identity of the presented patient and their partner. All couples were described as a serodifferent relationship with a partner who had HIV and was not virally suppressed. From a strictly epidemiological standpoint, HIV prevalence and incidence are higher among Black MSM and WSM compared to White MSM and WSM [1]. While it is true that many new HIV diagnoses are made among certain patient groups, this reduction to epidemiology ignores the individual risk factors that are present for a specific patient. Particularly in this setting where the fictional patient was seeking PrEP, it is evident that the patient felt they were at risk for HIV based on their partners’ HIV positive status. Implicit racism did not moderate these differences in assumed HIV risk indicating that medical students may require additional training about HIV risk-factors. Indeed, previous studies have underscored the variability and incompleteness of sexual history education in medical education [74, 75]. No effects of the interaction between patient and partner race were identified on assumed HIV risk.

### Implications

The results of the present investigation have multiple implications for training and public health interventions going forward with respect to efforts to reduce HIV. Studies have demonstrated mixed results with respect to changes in condom use during sex among people taking PrEP with some studies showing increases in condomless sex and others identifying no changes [36, 38, 39]. Notably, studies have identified increases in the frequency of condomless sex with partners who are known to be HIV positive [36, 38, 39]. Very little work has begun to explore whether PrEP is associated with changes in sexual behaviors among WSM or MSW, and our findings suggest MSW or WSM prescribed PrEP may be assumed by clinicians to change their sexual behaviors [39]. Regardless of whether these assumptions are accurate for any given patient, reduced condom use—actual or anticipated—is not a medically justifiable reason for a provider to withhold PrEP, a regimen that is extremely effective at preventing HIV even in the absence of condoms [2].

Previous work with prescribers has shown that MSM remain the group most likely to receive PrEP prescription [76–78]. Furthermore, most MSM patients currently prescribed PrEP also have a partner who is also taking PrEP [79]. While encouraging, this focuses attention on the need to identify barriers to PrEP prescription to patients in serodifferent relationships, such as the fictional couples presented in the current study including WSM and MSW, who also experience HIV risk when their sexual partner is HIV-positive and not virally suppressed. Sexual partners are often a source of support for patients’ decisions to begin taking PrEP in a serodifferent relationship [23, 24, 28, 80, 81]. One study found that when HIV positive people were educated about PrEP, they became advocates for encouraging their sexual partners to consider PrEP for HIV prevention [82].

Considering this support, it is critical that healthcare providers are prepared to engage patient couples in conversations about PrEP for HIV prevention given previously reported effectiveness of such counseling interventions [23, 24]. This is an important intervention, as research with MSM couples in which both partners are HIV-negative has shown low interest in PrEP given perceptions of low HIV risk, even in the setting of condomless sex with partners outside of the relationship [83]. Of the published resources for medical education regarding sexual health, all are focused on patients presenting without their sexual partner [84–89]. This indicates an opportunity for development of training materials, including standardized patient encounters including both a patient and their sexual partner for couples-based HIV risk-reduction counseling training. Additionally, training interventions that are focused on counseling skills for patients seeking PrEP are also needed. These interventions should include a specific focus on bias-free counseling and an emphasis on communication about the protective benefits of PrEP rather than avoidance of counseling based on assumptions of patient behavior if prescribed PrEP. Education about PrEP counseling is especially important now considering the updated CDC PrEP guidelines released in December 2021, which specify a patient request for PrEP as sufficient justification for prescription [90].

While the present study did identify effects of implicit racism, these effects were relatively limited and did not adversely affect judgment of Black patients. These findings are encouraging and may be explained by contemporary societal and educational trends. Recent research has found a population-level decline in implicit racism [91]. In the years since previous studies of medical students’ PrEP decision-making, there has been an increase in implicit bias training in medical education [70–72]. This may explain the lack of effects of implicit racism on the variables in this study as students may have been more aware of their vulnerability to make rash judgments about patients.

We did not assess medical students’ exposure to implicit bias training. However, previous research has identified mixed effects of implicit bias training on clinician behavior with respect to health disparities among groups affected by these biases [92]. This is an area of important future research to better understand how implicit biases affect assumptions about patients, and subsequently care decisions. This is particularly prudent given that many Black people report experiences of discrimination and racism during healthcare encounters [93–95]. A recent study found that over 30% of MSM reported experiencing discrimination when seeking PrEP [96]. Previous work has also found that MSM, particularly Black MSM, report experiencing discrimination by clinicians when seeking PrEP, including assumptions of promiscuity or heterosexist attitudes [97–101].

It is also important to consider the social context and current events occurring during the time this study was completed and that occurred following earlier studies of implicit biases and assumptions about patients seeking PrEP [50, 51] In the years since these studies, the Black Lives Matter movement for racial justice in the U.S. gained significant momentum and brought racial disparities into the public consciousness. This was further compounded by the high-profile killings of Black people by police officers in 2020, which occurred prior to initiation of this study. The COVID-19 pandemic, which disproportionately affected communities of color in the U.S., also brought attention to the health disparities experienced by these communities. It is important to consider these concurrently occurring sociopolitical events which may have affected the judgements study participants made about the fictional patients in this study; for example, hypervigilance about racial bias may help to explain the favorable assumptions about Black vs. White patients self-reported in the study despite accounts of racism reported by patients in real-world practice [93–95, 97–101].

### Limitations

There are several limitations of the present study that should be considered when interpreting the findings. First, all participants were medical students, so additional work is needed to determine generalizability of findings to practicing physicians. There has been a recent focus on medical students and medical education due to proximity to training, ease of access for study, and the need to ensure medical education prepares future clinicians to address disparities in HIV and PrEP prescription [102]. Still, it is important for future research to investigate assumptions about patients and implications for PrEP clinical decision-making among practicing clinicians as well to identify additional targets for improvement in training and practice.

Regarding the study sample, the limitation of geographic diversity should be acknowledged as the Southern U.S. was under-represented compared to the other regions. This may be an important sampling difference given that the Southern U.S. experiences a significant disparity in new HIV diagnoses and health professions education about PrEP has been shown to be less comprehensive in the South [1, 102] Furthermore, it should also be acknowledged that there is a slight unevenness in the distribution of participants between years in training, with a slight majority in the first and second years of training. For this reason, we controlled all analyses for participants’ year in medical education.

A related limitation is that the assumptions assessed in this study were not connected to patient outcomes or intention to prescribe PrEP to the patient presented. We opted to probe only the assumptions that students made about the patients presented in these vignettes to specifically identify patterns of differences in judgment of about various patients seeking PrEP in the context of a sexual relationship. Given medical students cannot practice independently, we felt that investigation of students’ assumptions about patients seeking PrEP was more appropriate and would provide more actionable targets for improving training about PrEP for future physicians.

Furthermore, some have critiqued the IAT, including whether its results are actually indicative of bias and whether these biases are actually connected to discriminatory action in the real-world, outside of experimental contexts [103, 104]. Theoretically, clinicians’ implicit biases may predispose them to make quick and often negative judgments about particular groups (e.g., racial minorities) [105–107]. Results of previous studies of relationships between clinicians’ implicit biases and patient outcomes and clinical interactions have been mixed [105, 107–111]. Some studies have found that implicit racial biases were associated with assumptions of medication non-adherence among Black patients, specifically for coronary artery thrombolysis and several pediatric conditions, like asthma [112–114]. The majority of studies have not identified a causal relationship between clinicians’ implicit biases and patient treatment outcomes, indicating that the effects of implicit biases may manifest in patient-provider communication rather than in an experimental setting [108, 109]. The IAT is also limited in its ability to assess intersectional stigma, as each version of the IAT assesses implicit attitudes towards one identity alone. For example, an IAT assesses attitudes towards Black people, men, or gay people individually rather than attitudes towards a Black gay man collectively. However, previous work has attempted to explore intersectional implicit attitudes through comparisons by marginalized status (eg. race) within another marginalized status (gay men).

An additional limitation is that our manipulation check item only asked about the race of the presented patient. Additional manipulation check items, such as asking about the gender identity of the patient, or about the race or gender identity of the patient’s partner for the relevant conditions may have potentially resulted in a sample that was more attuned to the systematic variation and thus more accurate results. In the replication of analyses, we found that mean assumed condomless sex and extra-relational sex was higher among the group of participants who failed the manipulation check compared to those who failed, however minimal differences with respect to analyses by patient and partner race. Finally, our vignettes presented a patient who explicitly requested a prescription for PrEP. This is likely a realistic scenario, given that conversations about PrEP are often initiated by the patient rather than the clinician, perhaps less so for non-MSM patients as this group still comprises a majority of PrEP prescriptions [115]. A future study may benefit from assessment of clinical decision-making for PrEP when the patient does not explicitly request a prescription to further elucidate mechanisms of bias affecting decision-making about PrEP prescription given the numerous, systemic barriers (eg. patient awareness of PrEP, self-assessment of HIV risk) that exist prior to a patient presenting to a clinician seeking PrEP. Finally, additional study is needed to investigate assumptions about patients identifying as transgender seeking PrEP given the HIV incidence disparities and lack of access to PrEP experienced by these patients [116–118].

## Conclusions

The present study explored the relationships between patient and sexual partner demographics (race, gender identity) and medical students’ assumptions about patients’ behavior if prescribed PrEP and HIV risk. In addition, we also examined the moderating role of implicit racism on the association between patient characteristics and participant assumptions about patient behavior and HIV risk to identify where additional training interventions are needed. Overall, we found minimal effects of patient race on assumptions about behavior in the setting of PrEP prescription as well as minimal moderating effects of implicit racism on these assumptions. Our findings are in line with previous studies exploring the role of implicit racism in assumptions about patient seeking PrEP and suggest that implicit racism may be present but not affecting judgements about fictional patients in an experimental setting. Considering this, our findings may bode positively for future efforts to improve prescription of PrEP to a greater diversity of patients when today’s medical students transition to independent practice. Further work is needed to ensure that medical education includes up to date and bias-free content about the HIV epidemic in the U.S., including population and individual-level risk factors for HIV, as well as strategies for effective couples-based HIV risk-reduction counseling.

## Supporting information

S1 Data (SAV)

S1 Table Demographics of the samples responding to each vignette. (PDF)

S2 Table Demographics by manipulation check status. (PDF)

S1 File Appendix 1. Study instrument.
(PDF)

S2 File Appendix 2. Replication of analyses with full sample.
(PDF)

## List of abbreviations

CIs: confidence intervals
HIV: human immunodeficiency virus
IAT: Implicit Association Test
MSM: men who have sex with men
MSW: men who have sex with women
PrEP: pre-exposure prophylaxis
TDF/FTC: emtricitabine/tenofovir disoproxil fumarate
WSM: women who have sex with men
