# Supplementary material for: Assumptions about patients seeking PrEP: Exploring the effects of patient and sexual partner race and gender identity and the moderating role of implicit racism
Source: PLoS One. 2022 Jul 1;17(7):e0270861. doi: 10.1371/journal.pone.0270861 (PMC9249206; doi:10.1371/journal.pone.0270861)
Supplement: S1 Table — (PDF) [file pone.0270861.s002.pdf]

SUPPLEMENTAL TABLE 1. Demographics of the samples responding to each vignette.

	1		2		3		4		5		6	
Couple Composition	**Pt: WM Partner: WM**		**Pt: BM Partner: WM**		**Pt: WM Partner: BM**		**Pt: BM Partner: BM**		**Pt: WF Partner: WM**		**Pt: WF Partner: BM**	
Sample Size	*n* = 125		*n* = 122		*n* = 123		*n* = 123		*n* = 124		*n* = 125	
**Academic Program**	***n***	**%**	***n***	**%**	***n***	**%**	***n***	**%**	***n***	**%**	***n***	**%**
Medicine (allopathic-MD)	70	56.0%	62	50.8%	62	50.4%	73	59.3%	68	54.8%	74	59.2%
Medicine (osteopathic-DO)	55	44.0%	60	49.2%	61	49.6%	50	40.7%	56	45.2%	51	40.8%
**Year of Training**												
1st	31	24.8%	24	19.7%	33	26.8%	36	29.3%	45	36.3%	42	33.6%
2nd	39	31.2%	43	35.2%	37	30.1%	29	23.6%	35	28.2%	27	21.6%
3rd	23	18.4%	32	26.2%	28	22.8%	30	24.4%	25	20.2%	25	20.0%
4th+	32	25.6%	23	18.9%	25	20.3%	28	22.8%	19	15.3%	31	24.8%
**Race/Ethnicity**												
White	73	58.4%	63	51.6%	67	54.5%	63	51.2%	63	50.8%	63	50.4%
Black	6	4.8%	4	3.3%	5	4.1%	5	4.1%	2	1.6%	4	3.2%
Hispanic/Latino	1	0.8%	6	4.9%	1	0.8%	12	9.8%	11	8.9%	2	1.6%
Asian	39	31.2%	35	28.7%	45	36.6%	34	27.6%	33	26.6%	44	35.2%
Other	6	4.8%	14	11.5%	5	4.1%	9	7.3%	15	12.1%	12	9.6%
**Sexual Orientation**												
Heterosexual (straight)	111	88.8%	109	89.3%	104	84.6%	109	88.6%	104	83.9%	112	89.6%
Homosexual (gay/lesbian)	5	4.0%	4	3.3%	9	7.3%	6	4.9%	6	4.8%	5	4.0%
Bisexual	7	5.6%	7	5.7%	8	6.5%	6	4.9%	14	11.3%	6	4.8%
Other	2	1.6%	2	1.6%	2	1.6%	2	1.6%	0	0.0%	2	1.6%
**Gender Identity**												
Man (cisgender male)	51	40.8%	52	42.6%	42	34.1%	62	50.4%	50	40.3%	53	42.4%
Woman (cisgender female)	74	59.2%	70	57.4%	79	64.2%	59	48.0%	72	58.1%	69	55.2%
Other	0	0.0%	0	0.0%	2	1.6%	2	1.6%	2	1.6%	3	2.4%
**Region**												
South	13	10.4%	9	7.4%	9	7.3%	8	6.5%	7	5.6%	9	7.2%
Northeast	28	22.4%	29	23.8%	27	22.0%	25	20.3%	24	19.4%	21	16.8%
West	31	24.8%	26	21.3%	22	17.9%	29	23.6%	28	22.6%	33	26.4%
Midwest	53	42.4%	58	47.5%	65	52.8%	61	49.6%	65	52.4%	62	49.6%
	M	***SD***	***M***	***SD***	***M***	***SD***	***M***	***SD***	***M***	***SD***	***M***	***SD***
Age	25.4	3.0	26.1	3.0	25.6	2.7	26.1	3.4	25.5	2.6	25.7	3.0
Racism *d*-Score	0.22	0.48	0.25	0.44	0.30	0.44	0.31	0.44	0.26	0.42	0.26	0.45

SUPPLEMENTAL TABLE 1. Demographics of the samples responding to each vignette (continued).

	7		8		9		10		11		12		
Couple Composition	**Pt: BF Partner: WM**		**Pt: BF Partner: BM**		**Pt: WM Partner: WF**		**Pt: WM Partner: BF**		**Pt: BM Partner: WF**		**Pt: BM Partner: BF**		
Sample Size	*n* = 125		*n* = 123		*n* =120		*n* = 123		*n* = 118		*n* = 121		
**Academic Program**	***n***	**%**	***n***	**%**	***n***	**%**	***n***	**%**	***n***	**%**	***n***	**%**	***p*[a]**
Medicine (allopathic-MD)	58	46.4%	65	52.8%	64	53.3%	65	52.8%	61	51.7%	66	54.5%	0.77
Medicine (osteopathic-DO)	67	53.6%	58	47.2%	56	46.7%	58	47.2%	57	48.3%	55	45.5%	
**Year of Training**													0.29
1st	44	35.2%	39	31.7%	30	25.0%	37	30.1%	28	23.7%	40	33.1%	
2nd	32	25.6%	29	23.6%	41	34.2%	42	34.1%	32	27.1%	38	31.4%	
3rd	26	20.8%	28	22.8%	20	16.7%	21	17.1%	25	21.2%	23	19.0%	
4th+	23	18.4%	27	22.0%	29	24.2%	23	18.7%	33	28.0%	20	16.5%	
**Race/Ethnicity**													**0.03**
White	69	55.2%	59	48.0%	69	57.5%	75	61.0%	73	61.9%	66	54.5%	
Black	3	2.4%	6	4.9%	5	4.2%	3	2.4%	5	4.2%	2	1.7%	
Hispanic/Latino	4	3.2%	3	2.4%	6	5.0%	7	5.7%	1	0.8%	7	5.8%	
Asian	38	30.4%	48	39.0%	33	27.5%	34	27.6%	33	28.0%	36	29.8%	
Other	11	8.8%	7	5.7%	7	5.8%	4	3.3%	6	5.1%	10	8.3%	
**Sexual Orientation**													0.81
Heterosexual (straight)	107	85.6%	108	87.8%	104	86.7%	104	84.6%	101	85.6%	107	88.4%	
Homosexual (gay/lesbian)	4	3.2%	5	4.1%	5	4.2%	8	6.5%	9	7.6%	3	2.5%	
Bisexual	9	7.2%	8	6.5%	8	6.7%	11	8.9%	7	5.9%	10	8.3%	
Other	5	4.0%	2	1.6%	3	2.5%	0	0.0%	1	0.8%	1	0.8%	
**Gender Identity**													**0.03**
Man (cisgender male)	60	48.0%	58	47.2%	53	44.2%	37	30.1%	46	39.0%	40	33.1%	
Woman (cisgender female)	64	51.2%	65	52.8%	63	52.5%	85	69.1%	72	61.0%	78	64.5%	
Other	1	0.8%	0	0.0%	4	3.3%	1	0.8%	0	0.0%	3	2.5%	
**Region**													0.98
South	6	4.8%	7	5.7%	9	7.5%	7	5.7%	10	8.5%	9	7.4%	
Northeast	26	20.8%	34	27.6%	26	21.7%	24	19.5%	23	19.5%	30	24.8%	
West	31	24.8%	31	25.2%	24	20.0%	32	26.0%	24	20.3%	21	17.4%	
Midwest	62	49.6%	51	41.5%	61	50.8%	60	48.8%	61	51.7%	61	50.4%	
	M	***SD***	***M***	***SD***	***M***	***SD***	***M***	***SD***	***M***	***SD***	***M***	***SD***	***p*[b]**
Age	25.4	2.3	25.9	3.1	25.3	2.5	25.4	2.7	25.8	3.2	25.0	2.1	0.06
Racism *d*-Score	0.33	0.46	0.35	0.40	0.26	0.44	0.25	0.43	0.28	0.42	0.31	0.39	0.46

a. *p*-value represents Fisher’s Exact Test comparison.

b. *p*-value represents analysis of variance (ANOVA) comparison.
