# Supplementary material for: Assumptions about patients seeking PrEP: Exploring the effects of patient and sexual partner race and gender identity and the moderating role of implicit racism
Source: PLoS One. 2022 Jul 1;17(7):e0270861. doi: 10.1371/journal.pone.0270861 (PMC9249206; doi:10.1371/journal.pone.0270861)
Supplement: S2 Table — (PDF) [file pone.0270861.s003.pdf]

SUPPLEMENTAL TABLE 2. Demographics by manipulation check status.

	Passed (*n* = 1,472)		Failed (*n* = 171)		
	n	**%**	***n***	**%**	p^a
**Academic Program**					0.51
Allopathic Medicine (MD)	788	53.5%	87	50.9%	
Osteopathic Medicine (DO)	684	46.5%	84	49.1%	
**Year of Training**					0.55
1st	429	29.1%	51	29.8%	
2nd	424	28.8%	41	24.0%	
3rd	306	20.8%	37	21.6%	
4th+	313	21.3%	42	24.6%	
**Race/Ethnicity**					0.20
White	803	54.6%	78	45.6%	
Black	50	3.4%	8	4.7%	
Hispanic/Latino	61	4.1%	11	6.4%	
Asian	452	30.7%	59	34.5%	
Other	106	7.2%	15	8.8%	
**Sexual Orientation**					**0.01**
Heterosexual (straight)	1,280	87.0%	161	94.2%	
Homosexual (gay/lesbian)	69	4.7%	3	1.8%	
Bisexual	101	6.9%	3	1.8%	
Other	22	1.5%	4	2.3%	
**Gender Identity**					0.89
Man (cisgender male)	604	41.0%	67	39.2%	
Woman (cisgender female)	850	57.7%	102	59.6%	
Other	18	1.2%	2	1.2%	
**Region**					0.71
South	103	7.0%	14	8.2%	
Northeast	317	21.5%	41	24.0%	
West	332	22.6%	40	23.4%	
Midwest	720	48.9%	76	44.4%	
	M	***SD***	***M***	***SD***	p^b
Age	25.6	2.83	26.0	3.85	0.16
Racism *d*-Score	0.28	0.44	0.34	0.41	0.11

a. *p*-value represents Fisher's Exact Test comparison.
b. *p*-value represents independent samples *t*-test comparison.
