# Supplementary material for: Assumptions about patients seeking PrEP: Exploring the effects of patient and sexual partner race and gender identity and the moderating role of implicit racism
Source: PLoS One. 2022 Jul 1;17(7):e0270861. doi: 10.1371/journal.pone.0270861 (PMC9249206; doi:10.1371/journal.pone.0270861)
Supplement: S1 File — Study instrument. (PDF) [file pone.0270861.s004.pdf]

RECRUITMENT MATERIALS

Initial message:

Dear Medical Student,

You are invited to participate in an educational research study about preventive medicine among various patient groups. You will be asked to review a patient vignette and answer follow up questions. The results will be used to improve clinical vignettes in medical education. You will have the option of receiving a debrief message following the completion of our study.

Immediately after completing the study, **you will receive a $10 gift card** as compensation for your time. We estimate participating in this study will take approximately 20 minutes. All responses will be **completely anonymous and confidential**, so please answer honestly.

CLICK THIS LINK to begin the study:
[*LINK INSERTED HERE*]

This study is being conducted by Samuel Bunting (MS4) and Sarah Garber, PhD, of the Chicago Medical School, and College of Pharmacy, respectively at Rosalind Franklin University in North Chicago, Illinois, USA. If you have questions or concerns about this survey, please contact Sarah Garber at sarah.garber@rosalindfranklin.edu or Samuel Bunting at samuel.bunting@my.rfums.org.

This study was approved by the Institutional Review Board of Rosalind Franklin University (Protocol: COP-20-256 on September 24, 2020).

Follow up message once a participant indicated interest:

Dear Medical Student,

Thank you for signing up to participate in this study related to preventive medicine in various patient populations! Please click the link below or copy and paste the URL below to be taken to the study portal to begin the study. Once you have finished the study, you will receive the gift card redemption instructions via email.

As a reminder, this study must be completed on a device with a physical keyboard. If you are not utilizing a device with a physical keyboard now, please come back to this study when you are using a device with a physical keyboard.

Follow this link to the Study Portal: [*LINK INSERTED HERE*]

Part 1: INFORMED CONSENT FOR RESEARCH PARTICIPATION

Evaluating Students' Perceptions of Preventive Medicine in Various Patient Groups

You are invited to be in a research study of students' perceptions of Preventive Medicine in Various Patient Groups. You were selected as a possible participant because you are a student in a health professions educational program in the United States. We ask that you read this form and ask any questions you may have before agreeing to be in the study.

This study is being conducted by: Samuel Bunting, MS4, Chicago Medical School, Rosalind Franklin University of Medicine and Science and Sarah Garber, PhD, College of Pharmacy, Rosalind Franklin University of Medicine and Science.

Procedures: If you agree to be in this study, we would ask you to do the following things:

- Complete a study instrument regarding about preventive medicine among various patient groups.
- The survey will take approximately 20 minutes and you will be asked to complete it once.

Confidentiality: The records of this study will be kept private. In any sort of report we might publish, we will not include any information that will make it possible to identify a subject. Research records will be stored securely and only researchers will have access to the records. No information that you submit as a part of the research activities or for the administration of the study completion incentive will be utilized to identify a specific respondent's responses. As data required for administration of the gift card incentive following cessation of the study is entered and maintained by Qualtrics, no member of the research team will have access to this information. Confidentiality will be maintained pursuant to the data protection policies of Qualtrics and any interface the RFUMS researchers have with this data will be protected by the encryption and security of the RFUMS network.

Voluntary Nature of the Study: Participation in this study is voluntary. Your decision whether or not to participate will not affect your current or future relations with Rosalind Franklin University. If you decide to participate, you are free to not answer any question or to withdraw at any time.

Contacts and Questions: Please ask any questions you have now. If you have questions later, you may contact Samuel Bunting at Samuel.bunting@my.rfums.org or 419-357-3607, or Sarah Garber, PhD at sarah.garber@rosalindfranklin.edu or at 847-578-8577. If you have any questions or concerns regarding your rights as a subject in this study, you may call the IRB office (phone: 847-578-8713 or email: IRB@rosalindfranklin.edu).

You may print a copy of this information to keep for your records.

This research study COP-20-256 received approval as an EXEMPT human subjects research project from the Rosalind Franklin University IRB on September 24, 2020.

Statement of Consent: I have read the above information and have received answers to any questions I asked. I consent to take part in the study.

- ◯ YES, I consent to participation.
- ◯ NO, I do NOT consent to participation.

Are you over 18 years of age?

- ◯ Yes
- ◯ No

This survey MUST be completed at a computer or other device with a PHYSICAL KEYBOARD. It CANNOT be completed from a mobile device. If you are not at a device with a physical keyboard, please close the survey and re-open the link from a device with a physical keyboard.

Are you using a device with a physical keyboard?

- ◯ Yes
- ◯ No

Part 2: VIGNETTE AND FOLLOW-UP

Thank you for agreeing to participate in this research study! We appreciate your time.

We will present a clinical case to you, followed by some specific follow up items related to the case. The clinical case represents a fictional patient in a fictional primary care office.

The reason for the patient's visit has been recorded by a medical assistant. Demographic information, including insurance coverage and pharmacy preferences are shown. The patient also had lab work done before this appointment, and the results are presented to the right of the patient summary.

VIGNETTE AND PATIENT AND/OR PATIENT AND PARTNER IMAGES PRESENTED HERE. SEE PAGES 11-17 IN THIS DOCUMENT, AND FIGURE 2 IN MANUSCRIPT FOR ALL PRESENTATIONS INCLUDING ALL IMAGES UTILIZED TO REPRESENT PATIENTS AND/OR PARTNERS.

1. Based on this information, is the patient a potential candidate for HIV Pre-Exposure Prophylaxis?

- ○ Yes
- ○ No

2. How likely is the patient to have more condomless sex if prescribed PrEP?

Extremely Unlikely (1)	Moderately Unlikely (2)	Slightly Unlikely (3)	Neither Likely nor Unlikely (4)	Slightly Likely (5)	Moderately Likely (6)	Extremely Likely (7)

3. How likely is the patient to have extra-relational sex if prescribed PrEP (ie. sex outside of their current relationship)?

Extremely Unlikely (1)	Moderately Unlikely (2)	Slightly Unlikely (3)	Neither Likely nor Unlikely (4)	Slightly Likely (5)	Moderately Likely (6)	Extremely Likely (7)

4. If prescribed PrEP, how likely is it that the patient would adhere to the medication?

Extremely Unlikely (1)	Moderately Unlikely (2)	Slightly Unlikely (3)	Neither Likely nor Unlikely (4)	Slightly Likely (5)	Moderately Likely (6)	Extremely Likely (7)

5. How high is this patient's overall HIV risk without PrEP?

Extremely Low (1)	Moderately Low (2)	Slightly Low (3)	Neither High nor Low (4)	Slightly High (5)	Moderately High (6)	Extremely High (7)

Part 3: IMPLICIT RACISM IAT

Here we present the materials, comprising the implicit racism implicit association test (IAT). The first group of images represent the screens participants progressed through while completing the IAT. The second section includes the visual and textual stimuli representing the images to convey White and Black people as well as the words used for positive and negative attributes. These materials were taken directly from the publicly available IAT library of Project Implicit.[1]

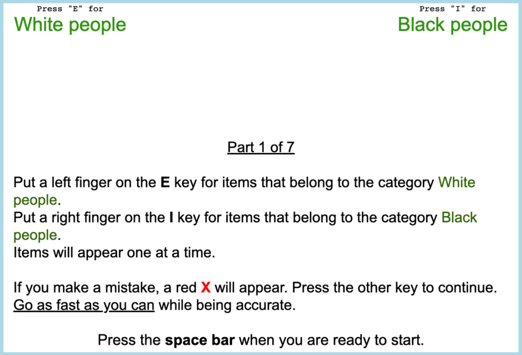

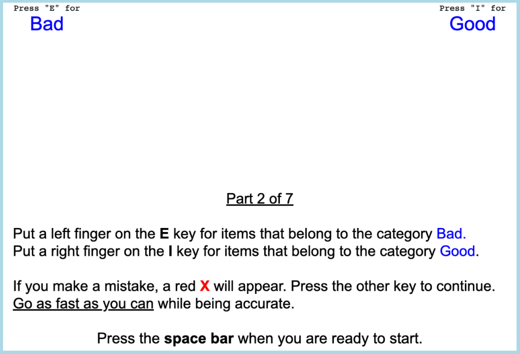

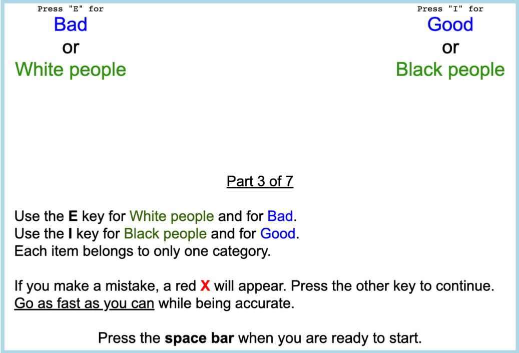

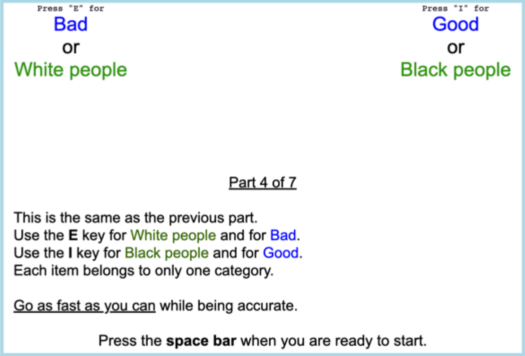

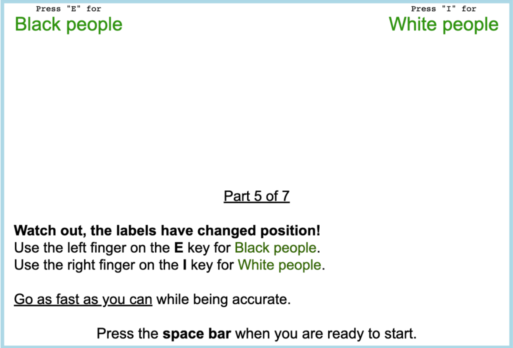

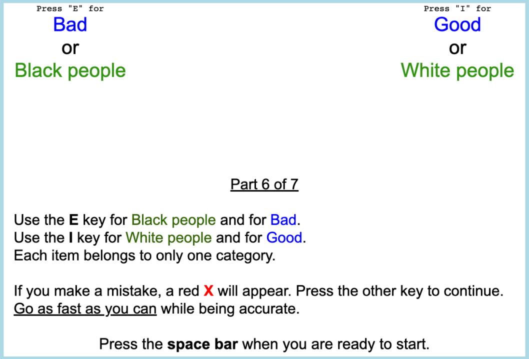

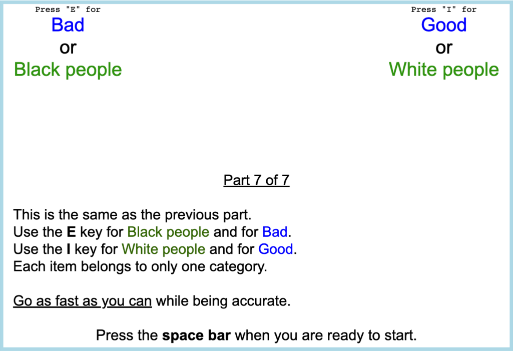

BLACK IMAGES:

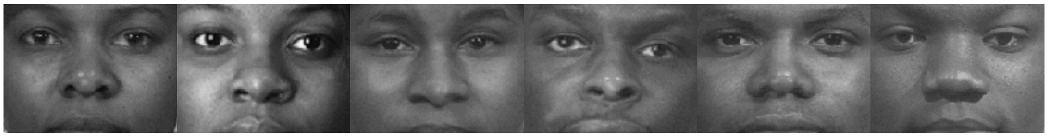

WHITE IMAGES:

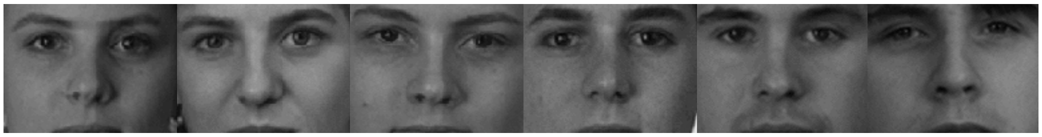

POSITIVE ATTRIBUTES:
Love, Cheer, Friend, Pleasure, Adore, Cheerful, Friendship, Joyful, Smiling, Cherish, Excellent, Glad, Joyous, Spectacular, Appealing, Delight, Excitement, Laughing, Attractive, Delightful, Fabulous, Glorious, Pleasing, Beautiful, Fantastic, Happy, Lovely, Terrific, Celebrate, Enjoy, Magnificent, Triumph

NEGATIVE ATTRIBUTES:
Abuse, Grief, Poison, Sadness, Pain, Despise, Failure, Nasty, Angry, Detest, Horrible, Negative, Ugly, Dirty, Gross, Evil, Rotten, Annoy, Disaster, Horrific, Scorn, Awful, Disgust, Hate, Humiliate, Selfish, Tragic, Bothersome, Hatred, Hurtful, Sickening, Yucky

[1] Bar-Anan Y. minnoJS File Library. https://www.jsdelivr.com/package/gh/baranan/minno-tasks. Published 2021. Accessed July 12, 2020.

Part 4: MANIPULATION CHECK ITEM

1. What was the race of the PATIENT presented in the clinical scenario at the beginning of this study?

- ○ Asian
- ○ Black
- ○ White

Part 5: DEMOGRAPHICS

The final group of items will ask some general demographic information.

1. Which academic program are you enrolled in?

- ○ Medicine (allopathic-MD)
- ○ Medicine (osteopathic-DO)

2. If other, please specify your academic program: ____________________

3. What is your academic standing in your program?

- ○ 1st year
- ○ 2nd year
- ○ 3rd year
- ○ 4th year
- ○ Other ____________________

4. Please indicate your age in years: ____________________

5. What is your degree of religiosity?

Not religious at all (1)	(2)	Neutral (3)	(4)	Very Religious (5)

6. Which of the following was your sex assigned at birth?

- ○ Male
- ○ Female

7. What is your gender identity?

- ○ Man (cisgender male)
- ○ Woman (cisgender female)
- ○ Transgender man
- ○ Transgender woman
- ○ Gender nonbinary
- ○ Other ______________________

8. What is your race/ethnicity?

- ○ African-American (Black)
- ○ Caucasian (White)
- ○ Hispanic/Latino
- ○ Native American
- ○ Asian
- ○ Other ______________________

9. Which of the following best describes your sexual orientation?

- ○ Heterosexual (straight)
- ○ Homosexual (gay)
- ○ Bisexual
- ○ Other ______________________

10. In which state do you attend school?

DROP DOWN MENU OF ALL 50 U.S. STATES

On the following pages, all the experimental stimuli (vignettes) are presented. These represent all combinations of patients and partners across races and gender identities representing MSM, MSW, and WSM.

Patient: Michael

Sex at Birth: Male
Gender Identity: Male

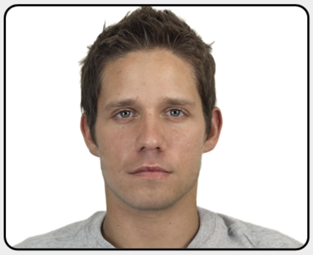

	Value	Reference
Glucose	90	70-110
Na^{+}	141	135-146
K^{+}	5.0	3.5-5.3
Cl^{-}	103	95-108
Ca^{2+}	9.4	8.5-10.3
BUN	12	9-20
Cr	0.8	0.55-1.25
AST	27	17-59
ALT	29	21-72

	Value
HIV Antibody/Antigen	Non-Reactive

Current Medications:
-Daily Multivitamin

Partner: Andrew

Sex at Birth: Male
Gender Identity: Male

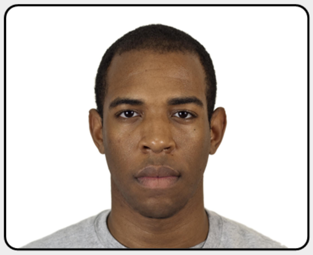

	Value	Reference
Glucose	84	70-110
Na^{+}	139	135-146
K^{+}	4.3	3.5-5.3
Cl^{-}	101	95-108
Ca^{2+}	9.1	8.5-10.3
BUN	10	9-20
Cr	0.7	0.55-1.25
AST	25	17-59
ALT	23	21-72

	Value	Reference
HIV Antibody/ Antigen	**REACTIVE**	
Viral Load	**547 copies/dL**	**<20**
Absolute CD4^{+} Count	**683 cells/uL**	**490-1740**

Current Medications:
-bictegravir-emtricitabine-tenofovir alafenamide 50-200-25 mg tablet; take one tablet by mouth daily

Reason for visit:

Michael is a 29 year old presenting for an annual physical and is accompanied by his partner, Andrew. The patient has recently begun a new relationship with Andrew.

Andrew was diagnosed with HIV and has started taking antiretroviral therapy but is not virally suppressed.

Michael mentions seeing a YouTube commercial for HIV Pre-Exposure Prophylaxis (PrEP) and asks if this would be something he should consider.

He reports social alcohol use, occasionally drinking 2-3 beers with friends on the weekends and denies tobacco, marijuana, or other drug use. The patient takes no medications other than a multivitamin. Family, medical, and surgical history are non-contributory. He has health insurance through his employer.

Patient: Michael

Sex at Birth: Male
Gender Identity: Male

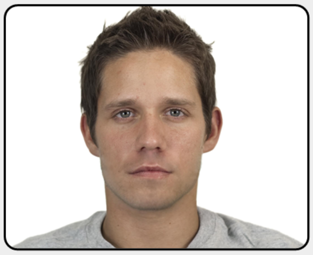

	Value	Reference
Glucose	90	70-110
Na^{+}	141	135-146
K^{+}	5.0	3.5-5.3
Cl^{-}	103	95-108
Ca^{2+}	9.4	8.5-10.3
BUN	12	9-20
Cr	0.8	0.55-1.25
AST	27	17-59
ALT	29	21-72

	Value
HIV Antibody/Antigen	Non-Reactive

Current Medications:
-Daily Multivitamin

Partner: Andrew

Sex at Birth: Male
Gender Identity: Male

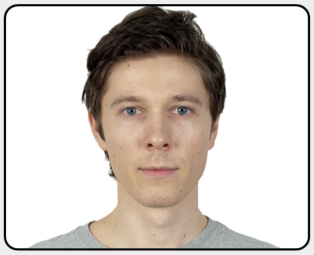

	Value	Reference
Glucose	84	70-110
Na^{+}	139	135-146
K^{+}	4.3	3.5-5.3
Cl^{-}	101	95-108
Ca^{2+}	9.1	8.5-10.3
BUN	10	9-20
Cr	0.7	0.55-1.25
AST	25	17-59
ALT	23	21-72

	Value	Reference
HIV Antibody/ Antigen	**REACTIVE**	
Viral Load	**547 copies/dL**	**<20**
Absolute CD4^{+} Count	**683 cells/uL**	**490-1740**

Current Medications:
-bictegravir-emtricitabine-tenofovir alafenamide 50-200-25 mg tablet; take one tablet by mouth daily

Reason for visit:

Michael is a 29 year old presenting for an annual physical and is accompanied by his partner, Andrew. The patient has recently begun a new relationship with Andrew.

Andrew was diagnosed with HIV and has started taking antiretroviral therapy but is not virally suppressed.

Michael mentions seeing a YouTube commercial for HIV Pre-Exposure Prophylaxis (PrEP) and asks if this would be something he should consider.

He reports social alcohol use, occasionally drinking 2-3 beers with friends on the weekends and denies tobacco, marijuana, or other drug use. The patient takes no medications other than a multivitamin. Family, medical, and surgical history are non-contributory. He has health insurance through his employer.

Patient: Michael

Sex at Birth: Male
Gender Identity: Male

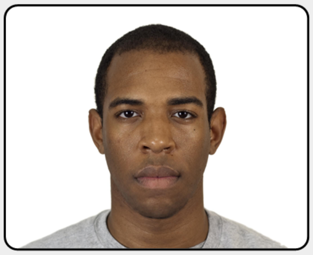

	Value	Reference
Glucose	90	70-110
Na^+	141	135-146
K^+	5.0	3.5-5.3
Cl^-	103	95-108
Ca^{2+}	9.4	8.5-10.3
BUN	12	9-20
Cr	0.8	0.55-1.25
AST	27	17-59
ALT	29	21-72

	Value
HIV Antibody/Antigen	Non-Reactive

Current Medications:
-Daily Multivitamin

Partner: Andrew

Sex at Birth: Male
Gender Identity: Male

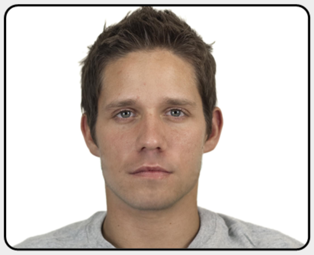

	Value	Reference
Glucose	84	70-110
Na^+	139	135-146
K^+	4.3	3.5-5.3
Cl^-	101	95-108
Ca^{2+}	9.1	8.5-10.3
BUN	10	9-20
Cr	0.7	0.55-1.25
AST	25	17-59
ALT	23	21-72

	Value	Reference
HIV Antibody/ Antigen	**REACTIVE**	
Viral Load	**547 copies/dL**	**<20**
Absolute CD4$^+$ Count	**683 cells/uL**	**490-1740**

Current Medications:
-bictegravir-emtricitabine-tenofovir alafenamide 50-200-25 mg tablet; take one tablet by mouth daily

Reason for visit:

Michael is a 29 year old presenting for an annual physical and is accompanied by his partner, Andrew. The patient has recently begun a new relationship with Andrew.

Andrew was diagnosed with HIV and has started taking antiretroviral therapy but is not virally suppressed.

Michael mentions seeing a YouTube commercial for HIV Pre-Exposure Prophylaxis (PrEP) and asks if this would be something he should consider.

He reports social alcohol use, occasionally drinking 2-3 beers with friends on the weekends and denies tobacco, marijuana, or other drug use. The patient takes no medications other than a multivitamin. Family, medical, and surgical history are non-contributory. He has health insurance through his employer.

Patient: Michael

Sex at Birth: Male
Gender Identity: Male

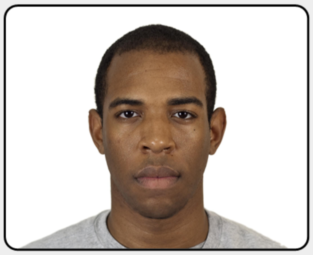

	Value	Reference
Glucose	90	70-110
Na^+	141	135-146
K^+	5.0	3.5-5.3
Cl^-	103	95-108
Ca^{2+}	9.4	8.5-10.3
BUN	12	9-20
Cr	0.8	0.55-1.25
AST	27	17-59
ALT	29	21-72

	Value
HIV Antibody/Antigen	Non-Reactive

Current Medications:
-Daily Multivitamin

Partner: Andrew

Sex at Birth: Male
Gender Identity: Male

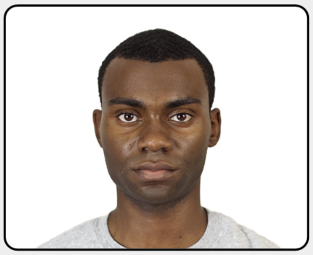

	Value	Reference
Glucose	84	70-110
Na^+	139	135-146
K^+	4.3	3.5-5.3
Cl^-	101	95-108
Ca^{2+}	9.1	8.5-10.3
BUN	10	9-20
Cr	0.7	0.55-1.25
AST	25	17-59
ALT	23	21-72

	Value	Reference
HIV Antibody/ Antigen	**REACTIVE**	
Viral Load	**547 copies/dL**	**<20**
Absolute CD4$^+$ Count	**683 cells/uL**	**490-1740**

Current Medications:
-bictegravir-emtricitabine-tenofovir alafenamide 50-200-25 mg tablet; take one tablet by mouth daily

Reason for visit:

Michael is a 29 year old presenting for an annual physical and is accompanied by his partner, Andrew. The patient has recently begun a new relationship with Andrew.

Andrew was diagnosed with HIV and has started taking antiretroviral therapy but is not virally suppressed.

Michael mentions seeing a YouTube commercial for HIV Pre-Exposure Prophylaxis (PrEP) and asks if this would be something he should consider.

He reports social alcohol use, occasionally drinking 2-3 beers with friends on the weekends and denies tobacco, marijuana, or other drug use. The patient takes no medications other than a multivitamin. Family, medical, and surgical history are non-contributory. He has health insurance through his employer.

Patient: Michael

Sex at Birth: Male
Gender Identity: Male

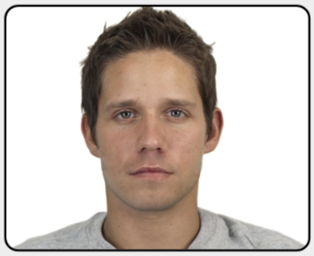

	Value	Reference
Glucose	90	70-110
Na^+	141	135-146
K^+	5.0	3.5-5.3
Cl^-	103	95-108
Ca^{2+}	9.4	8.5-10.3
BUN	12	9-20
Cr	0.8	0.55-1.25
AST	27	17-59
ALT	29	21-72

	Value
HIV Antibody/Antigen	Non-Reactive

Current Medications:
-Daily Multivitamin

Partner: Andrea

Sex at Birth: Female
Gender Identity: Female

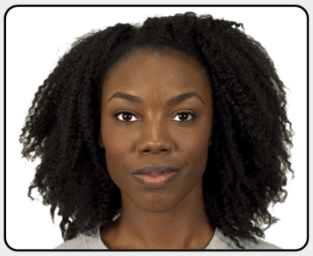

	Value	Reference
Glucose	84	70-110
Na^+	139	135-146
K^+	4.3	3.5-5.3
Cl^-	101	95-108
Ca^{2+}	9.1	8.5-10.3
BUN	10	9-20
Cr	0.7	0.55-1.25
AST	25	17-59
ALT	23	21-72

	Value	Reference
HIV Antibody/ Antigen	**REACTIVE**	
Viral Load	**547 copies/dL**	**<20**
Absolute $CD4^+$ Count	**683 cells/uL**	**490-1740**

Current Medications:
-bictegravir-emtricitabine-tenofovir alafenamide 50-200-25 mg tablet; take one tablet by mouth daily

Reason for visit:

Michael is a 29 year old presenting for an annual physical and is accompanied by his partner, Andrea. The patient has recently begun a new relationship with Andrea.

Andrea was diagnosed with HIV and has started taking antiretroviral therapy but is not virally suppressed.

Michael mentions seeing a YouTube commercial for HIV Pre-Exposure Prophylaxis (PrEP) and asks if this would be something he should consider.

He reports social alcohol use, occasionally drinking 2-3 beers with friends on the weekends and denies tobacco, marijuana, or other drug use. The patient takes no medications other than a multivitamin. Family, medical, and surgical history are non-contributory. He has health insurance through his employer.

Patient: Michael

Sex at Birth: Male
Gender Identity: Male

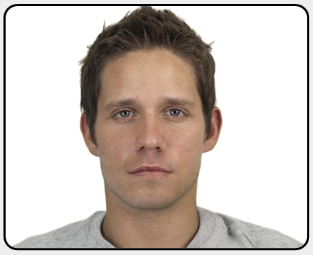

	Value	Reference
Glucose	90	70-110
Na^+	141	135-146
K^+	5.0	3.5-5.3
Cl^-	103	95-108
Ca^{2+}	9.4	8.5-10.3
BUN	12	9-20
Cr	0.8	0.55-1.25
AST	27	17-59
ALT	29	21-72

	Value
HIV Antibody/Antigen	Non-Reactive

Current Medications:
-Daily Multivitamin

Partner: Andrea

Sex at Birth: Female
Gender Identity: Female

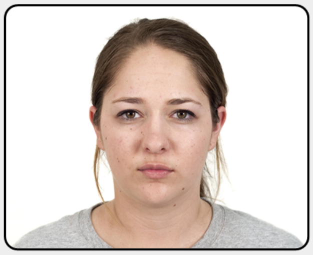

	Value	Reference
Glucose	84	70-110
Na^+	139	135-146
K^+	4.3	3.5-5.3
Cl^-	101	95-108
Ca^{2+}	9.1	8.5-10.3
BUN	10	9-20
Cr	0.7	0.55-1.25
AST	25	17-59
ALT	23	21-72

	Value	Reference
HIV Antibody/ Antigen	**REACTIVE**	
Viral Load	**547 copies/dL**	**<20**
Absolute $CD4^+$ Count	**683 cells/uL**	**490-1740**

Current Medications:
-bictegravir-emtricitabine-tenofovir alafenamide 50-200-25 mg tablet; take one tablet by mouth daily

Reason for visit:

Michael is a 29 year old presenting for an annual physical and is accompanied by his partner, Andrea. The patient has recently begun a new relationship with Andrea.

Andrea was diagnosed with HIV and has started taking antiretroviral therapy but is not virally suppressed.

Michael mentions seeing a YouTube commercial for HIV Pre-Exposure Prophylaxis (PrEP) and asks if this would be something he should consider.

He reports social alcohol use, occasionally drinking 2-3 beers with friends on the weekends and denies tobacco, marijuana, or other drug use. The patient takes no medications other than a multivitamin. Family, medical, and surgical history are non-contributory. He has health insurance through his employer.

Patient: Michael

Sex at Birth: Male
Gender Identity: Male

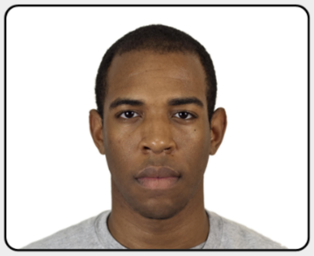

	Value	Reference
Glucose	90	70-110
Na^+	141	135-146
K^+	5.0	3.5-5.3
Cl^-	103	95-108
Ca^{2+}	9.4	8.5-10.3
BUN	12	9-20
Cr	0.8	0.55-1.25
AST	27	17-59
ALT	29	21-72

	Value
HIV Antibody/Antigen	Non-Reactive

Current Medications:
-Daily Multivitamin

Partner: Andrea

Sex at Birth: Female
Gender Identity: Female

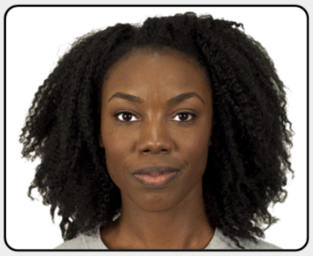

	Value	Reference
Glucose	84	70-110
Na^+	139	135-146
K^+	4.3	3.5-5.3
Cl^-	101	95-108
Ca^{2+}	9.1	8.5-10.3
BUN	10	9-20
Cr	0.7	0.55-1.25
AST	25	17-59
ALT	23	21-72

	Value	Reference
HIV Antibody/ Antigen	**REACTIVE**	
Viral Load	**547 copies/dL**	**<20**
Absolute $CD4^+$ Count	**683 cells/uL**	**490-1740**

Current Medications:
-bictegravir-emtricitabine-tenofovir alafenamide 50-200-25 mg tablet; take one tablet by mouth daily

Reason for visit:

Michael is a 29 year old presenting for an annual physical and is accompanied by his partner, Andrea. The patient has recently begun a new relationship with Andrea.

Andrea was diagnosed with HIV and has started taking antiretroviral therapy but is not virally suppressed.

Michael mentions seeing a YouTube commercial for HIV Pre-Exposure Prophylaxis (PrEP) and asks if this would be something he should consider.

He reports social alcohol use, occasionally drinking 2-3 beers with friends on the weekends and denies tobacco, marijuana, or other drug use. The patient takes no medications other than a multivitamin. Family, medical, and surgical history are non-contributory. He has health insurance through his employer.

Patient: Michael

Sex at Birth: Male
Gender Identity: Male

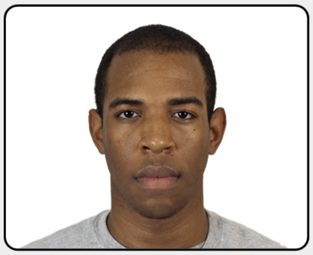

	Value	Reference
Glucose	90	70-110
Na^+	141	135-146
K^+	5.0	3.5-5.3
Cl^-	103	95-108
Ca^{2+}	9.4	8.5-10.3
BUN	12	9-20
Cr	0.8	0.55-1.25
AST	27	17-59
ALT	29	21-72

	Value
HIV Antibody/Antigen	Non-Reactive

Current Medications:
-Daily Multivitamin

Partner: Andrea

Sex at Birth: Female
Gender Identity: Female

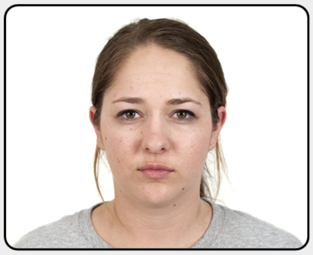

	Value	Reference
Glucose	84	70-110
Na^+	139	135-146
K^+	4.3	3.5-5.3
Cl^-	101	95-108
Ca^{2+}	9.1	8.5-10.3
BUN	10	9-20
Cr	0.7	0.55-1.25
AST	25	17-59
ALT	23	21-72

	Value	Reference
HIV Antibody/ Antigen	**REACTIVE**	
Viral Load	**547 copies/dL**	**<20**
Absolute $CD4^+$ Count	**683 cells/uL**	**490-1740**

Current Medications:
-bictegravir-emtricitabine-tenofovir alafenamide 50-200-25 mg tablet; take one tablet by mouth daily

Reason for visit:

Michael is a 29 year old presenting for an annual physical and is accompanied by his partner, Andrea. The patient has recently begun a new relationship with Andrea.

Andrea was diagnosed with HIV and has started taking antiretroviral therapy but is not virally suppressed.

Michael mentions seeing a YouTube commercial for HIV Pre-Exposure Prophylaxis (PrEP) and asks if this would be something he should consider.

He reports social alcohol use, occasionally drinking 2-3 beers with friends on the weekends and denies tobacco, marijuana, or other drug use. The patient takes no medications other than a multivitamin. Family, medical, and surgical history are non-contributory. He has health insurance through his employer.

<u>Patient</u>: Michelle

Sex at Birth: Female
Gender Identity: Female

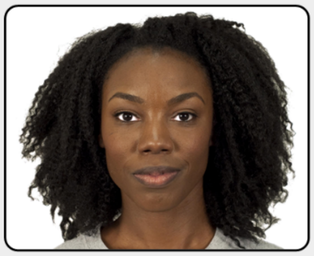

	Value	Reference
Glucose	90	70-110
Na^+	141	135-146
K^+	5.0	3.5-5.3
Cl^-	103	95-108
Ca^{2+}	9.4	8.5-10.3
BUN	12	9-20
Cr	0.8	0.55-1.25
AST	27	17-59
ALT	29	21-72

	Value
HIV Antibody/Antigen	Non-Reactive

Current Medications:
-Daily Multivitamin

<u>Partner</u>: Andrew

Sex at Birth: Male
Gender Identity: Male

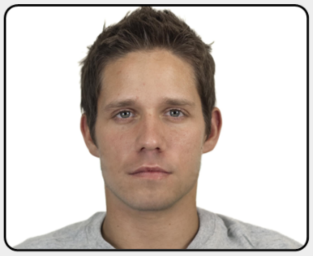

	Value	Reference
Glucose	84	70-110
Na^+	139	135-146
K^+	4.3	3.5-5.3
Cl^-	101	95-108
Ca^{2+}	9.1	8.5-10.3
BUN	10	9-20
Cr	0.7	0.55-1.25
AST	25	17-59
ALT	23	21-72

	Value	Reference
HIV Antibody/ Antigen	**REACTIVE**	
Viral Load	**547 copies/dL**	**<20**
Absolute $CD4^+$ Count	**683 cells/uL**	**490-1740**

Current Medications:
-bictegravir-emtricitabine-tenofovir alafenamide 50-200-25 mg tablet; take one tablet by mouth daily

<u>Reason for visit</u>:

<u>Michelle</u> is a 29 year old presenting for an annual physical and is accompanied by her partner, <u>Andrew</u>. The patient has recently begun a new relationship with <u>Andrew</u>.

<u>Andrew</u> was diagnosed with HIV and has started taking antiretroviral therapy but is not virally suppressed.

<u>Michelle</u> mentions seeing a YouTube commercial for HIV Pre-Exposure Prophylaxis (PrEP) and asks if this would be something she should consider.

She reports social alcohol use, occasionally drinking 2-3 beers with friends on the weekends and denies tobacco, marijuana, or other drug use. The patient takes no medications other than a multivitamin. Family, medical, and surgical history are non-contributory. She has health insurance through his employer.

<u>Patient</u>: Michelle

Sex at Birth: Female
Gender Identity: Female

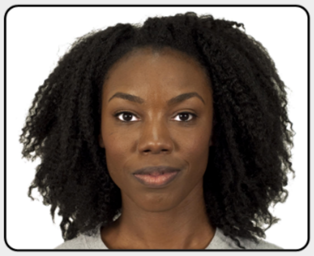

	Value	Reference
Glucose	90	70-110
Na^+	141	135-146
K^+	5.0	3.5-5.3
Cl^-	103	95-108
Ca^{2+}	9.4	8.5-10.3
BUN	12	9-20
Cr	0.8	0.55-1.25
AST	27	17-59
ALT	29	21-72

	Value
HIV Antibody/Antigen	Non-Reactive

Current Medications:
-Daily Multivitamin

<u>Partner</u>: Andrew

Sex at Birth: Male
Gender Identity: Male

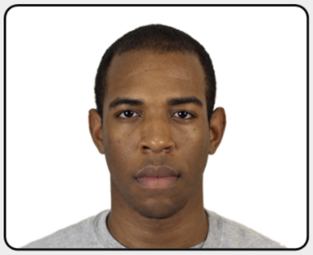

	Value	Reference
Glucose	84	70-110
Na^+	139	135-146
K^+	4.3	3.5-5.3
Cl^-	101	95-108
Ca^{2+}	9.1	8.5-10.3
BUN	10	9-20
Cr	0.7	0.55-1.25
AST	25	17-59
ALT	23	21-72

	Value	Reference
HIV Antibody/ Antigen	**REACTIVE**	
Viral Load	**547 copies/dL**	**<20**
Absolute $CD4^+$ Count	**683 cells/uL**	**490-1740**

Current Medications:
-bictegravir-emtricitabine-tenofovir alafenamide 50-200-25 mg tablet; take one tablet by mouth daily

<u>Reason for visit</u>:

<u>Michelle</u> is a 29 year old presenting for an annual physical and is accompanied by her partner, <u>Andrew</u>. The patient has recently begun a new relationship with <u>Andrew</u>.

<u>Andrew</u> was diagnosed with HIV and has started taking antiretroviral therapy but is not virally suppressed.

<u>Michelle</u> mentions seeing a YouTube commercial for HIV Pre-Exposure Prophylaxis (PrEP) and asks if this would be something she should consider.

She reports social alcohol use, occasionally drinking 2-3 beers with friends on the weekends and denies tobacco, marijuana, or other drug use. The patient takes no medications other than a multivitamin. Family, medical, and surgical history are non-contributory. She has health insurance through his employer.

Patient: Michelle

Sex at Birth: Female
Gender Identity: Female

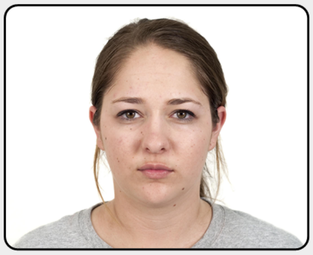

	Value	Reference
Glucose	90	70-110
Na^+	141	135-146
K^+	5.0	3.5-5.3
Cl^-	103	95-108
Ca^{2+}	9.4	8.5-10.3
BUN	12	9-20
Cr	0.8	0.55-1.25
AST	27	17-59
ALT	29	21-72

	Value
HIV Antibody/Antigen	Non-Reactive

Current Medications:
-Daily Multivitamin

Partner: Andrew

Sex at Birth: Male
Gender Identity: Male

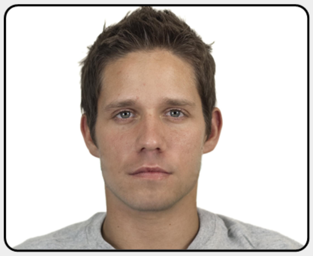

	Value	Reference
Glucose	84	70-110
Na^+	139	135-146
K^+	4.3	3.5-5.3
Cl^-	101	95-108
Ca^{2+}	9.1	8.5-10.3
BUN	10	9-20
Cr	0.7	0.55-1.25
AST	25	17-59
ALT	23	21-72

	Value	Reference
HIV Antibody/ Antigen	**REACTIVE**	
Viral Load	**547 copies/dL**	**<20**
Absolute $CD4^+$ Count	**683 cells/uL**	**490-1740**

Current Medications:
-bictegravir-emtricitabine-tenofovir alafenamide 50-200-25 mg tablet; take one tablet by mouth daily

Reason for visit:

Michelle is a 29 year old presenting for an annual physical and is accompanied by her partner, Andrew. The patient has recently begun a new relationship with Andrew.

Andrew was diagnosed with HIV and has started taking antiretroviral therapy but is not virally suppressed.

Michelle mentions seeing a YouTube commercial for HIV Pre-Exposure Prophylaxis (PrEP) and asks if this would be something she should consider.

She reports social alcohol use, occasionally drinking 2-3 beers with friends on the weekends and denies tobacco, marijuana, or other drug use. The patient takes no medications other than a multivitamin. Family, medical, and surgical history are non-contributory. She has health insurance through his employer.

Patient: Michelle

Sex at Birth: Female
Gender Identity: Female

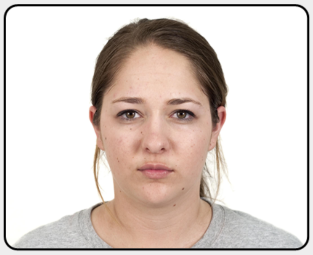

	Value	Reference
Glucose	90	70-110
Na^+	141	135-146
K^+	5.0	3.5-5.3
Cl^-	103	95-108
Ca^{2+}	9.4	8.5-10.3
BUN	12	9-20
Cr	0.8	0.55-1.25
AST	27	17-59
ALT	29	21-72

	Value
HIV Antibody/Antigen	Non-Reactive

Current Medications:
-Daily Multivitamin

Partner: Andrew

Sex at Birth: Male
Gender Identity: Male

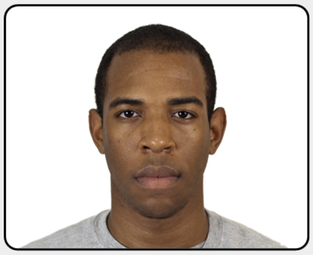

	Value	Reference
Glucose	84	70-110
Na^+	139	135-146
K^+	4.3	3.5-5.3
Cl^-	101	95-108
Ca^{2+}	9.1	8.5-10.3
BUN	10	9-20
Cr	0.7	0.55-1.25
AST	25	17-59
ALT	23	21-72

	Value	Reference
HIV Antibody/ Antigen	**REACTIVE**	
Viral Load	**547 copies/dL**	**<20**
Absolute $CD4^+$ Count	**683 cells/uL**	**490-1740**

Current Medications:
-bictegravir-emtricitabine-tenofovir alafenamide 50-200-25 mg tablet; take one tablet by mouth daily

Reason for visit:

Michelle is a 29 year old presenting for an annual physical and is accompanied by her partner, Andrew. The patient has recently begun a new relationship with Andrew.

Andrew was diagnosed with HIV and has started taking antiretroviral therapy but is not virally suppressed.

Michelle mentions seeing a YouTube commercial for HIV Pre-Exposure Prophylaxis (PrEP) and asks if this would be something she should consider.

She reports social alcohol use, occasionally drinking 2-3 beers with friends on the weekends and denies tobacco, marijuana, or other drug use. The patient takes no medications other than a multivitamin. Family, medical, and surgical history are non-contributory. She has health insurance through his employer.
