# Supplementary material for: Assumptions about patients seeking PrEP: Exploring the effects of patient and sexual partner race and gender identity and the moderating role of implicit racism
Source: PLoS One. 2022 Jul 1;17(7):e0270861. doi: 10.1371/journal.pone.0270861 (PMC9249206; doi:10.1371/journal.pone.0270861)
Supplement: S2 File — Replication of analyses with full sample. (PDF) [file pone.0270861.s005.pdf]

Appendix 2 - Table 1. Assumption means compared between manipulation check status.

	Anticipated Condomless Sex		Anticipated Extra-Relational Sex		Anticipated Non-Adherence		Assumed HIV Risk	
**MSM**								
	M (95%CI)	*p*	*M* (95%CI)	*p*	*M* (95%CI)	*p*	*M* (95%CI)	*p*
FAIL	4.85 (4.56, 5.15)	-	4.31 (4.03, 4.60)	-	2.73 (2.47, 3.00)	-	5.51 (5.20, 5.82)	-
PASS	4.73 (4.63, 4.83)	.45	3.83 (3.73, 3.92)	**<.001**	2.22 (2.13, 2.31)	**<.001**	5.59 (5.48, 5.70)	.64
**MSW**								
FAIL	5.14 (4.84, 5.45)	-	4.22 (3.94, 4.49)	-	2.48 (2.22, 2.73)	-	5.28 (4.98, 5.59)	-
PASS	4.77 (4.66, 4.88)	**.02**	3.82 (3.72, 3.92)	**.008**	2.26 (2.17, 2.35)	.12	5.53 (5.42, 5.64)	.14
**WSM**								
FAIL	4.67 (4.27, 5.07)	-	3.56 (3.21, 3.91)	-	2.74 (2.42, 3.06)	-	5.28 (4.91, 5.64)	-
PASS	4.67 (4.56, 4.79)	.99	3.83 (3.73, 3.93)	.15	2.2 (2.11, 2.29)	**.001**	5.50 (5.39, 5.60)	.26

Comparison of mean values for assumptions between the groups of participants who passed or failed the manipulation check item. Comparisons were conducted via ANCOVA controlling for respondents' year in training, sexual orientation, gender identity, and race. MSM = men who have sex with men; WSM = women who have sex with men; MSW = men who have sex with women.

Appendix 2 - Table 2. Main effects of patient and partner race on assumptions (full analytic sample, *N* = 1,643).

		Anticipated Condomless Sex		Anticipated Extra-Relational Sex		Anticipated Non-Adherence		Assumed HIV Risk	
**MSM**									
		M (95%CI)	*p*	*M* (95%CI)	*p*	*M* (95%CI)	*p*	*M* (95%CI)	*p*
	OVERALL	4.74 (4.64, 4.84)	-	3.88 (3.78, 3.98)	-	2.27 (2.19, 2.36)	-	5.58 (5.48, 5.68)	-
Patient Race	White	4.77 (4.63, 4.91)	*Ref.*	3.89 (3.76, 4.02)	*Ref.*	2.29 (2.17, 2.41)	*Ref.*	5.56 (5.42, 5.71)	*Ref.*
	Black	4.72 (4.58, 4.85)	.61	3.87 (3.74, 4.00)	.81	2.26 (2.14, 2.38)	.71	5.60 (5.46, 5.74)	.76
Partner Race	White	4.73 (4.59, 4.87)	*Ref.*	3.82 (3.69, 3.95)	*Ref.*	2.17 (2.05, 2.29)	*Ref.*	5.69 (5.55, 5.84)	*Ref.*
	Black	4.76 (4.62, 4.89)	.79	3.94 (3.81, 4.07)	.22	2.38 (2.25, 2.50)	**.02**	5.47 (5.32, 5.61)	**.03**
Pt. Race X Partner Race	White X White	4.69 (4.49, 4.89)	*Ref.*	3.94 (3.75, 4.13)	*Ref.*	2.34 (2.16, 2.52)	*Ref.*	5.51 (5.31, 5.72)	*Ref.*
	White X Black	4.75 (4.56, 4.93)	.69	3.80 (3.62, 3.97)	.28	2.17 (2.01, 2.34)[a1]	.18	5.68 (5.49, 5.87)	.24
	Black X White	4.82 (4.63, 5.01)	.35	3.94 (3.75, 4.12)	.98	2.41 (2.24, 2.59)[a1,a2]	.55	5.42 (5.22, 5.62)	.54
	Black X Black	4.71 (4.51, 4.92)	.86	3.84 (3.65, 4.04)	.50	2.17 (1.99, 2.35)[a2]	.18	5.71 (5.50, 5.92)	.20
**MSW**									
		M (95%CI)	*p*	*M* (95%CI)	*p*	*M* (95%CI)	*p*	*M* (95%CI)	*p*
	OVERALL	4.81 (4.71, 4.91)	-	3.86 (3.77, 3.95)	-	2.28 (2.19, 2.36)	-	5.51 (5.41, 5.61)	-
Patient Race	White	4.85 (4.70, 5.00)	*Ref.*	3.93 (3.80, 4.07)	*Ref.*	2.37 (2.25, 2.49)	*Ref.*	5.43 (5.28, 5.58)	*Ref.*
	Black	4.77 (4.63, 4.91)	.46	3.80 (3.67, 3.92)	.14	2.20 (2.08, 2.32)	**.05**	5.57 (5.43, 5.71)	.17
Partner Race	White	4.78 (4.63, 4.92)	*Ref.*	3.93 (3.80, 4.06)	*Ref.*	2.33 (2.21, 2.45)	*Ref.*	5.46 (5.31, 5.60)	*Ref.*
	Black	4.84 (4.70, 4.99)	.52	3.80 (3.67, 3.94)	.19	2.24 (2.12, 2.36)	.27	5.54 (5.40, 5.69)	.42
Pt. Race X Partner Race	White X White	4.77 (4.57, 4.98)	*Ref.*	3.71 (3.52, 3.90)	*Ref.*	2.19 (2.01, 2.36)	*Ref.*	5.58 (5.37, 5.79)	*Ref.*
	White X Black	4.77 (4.58, 4.96)	.98	3.88 (3.71, 4.05)	.19	2.21 (2.06, 2.37)[a]	.82	5.56 (5.37, 5.75)	.89
	Black X White	4.91 (4.71, 5.12)	.34	3.89 (3.71, 4.08)	.17	2.29 (2.12, 2.46)	.41	5.50 (5.30, 5.71)	.59
	Black X Black	4.78 (4.57, 5.00)	.95	3.97 (3.78, 4.17)	.06	2.45 (2.28, 2.63)[a]	**.04**	5.35 (5.14, 5.57)	.13
**WSM**									
		M (95%CI)	*p*	*M* (95%CI)	*p*	*M* (95%CI)	*p*	*M* (95%CI)	*p*
	OVERALL	4.67 (4.56, 4.78)	-	3.80 (3.70, 3.90)	-	2.24 (2.15, 2.32)	-	5.47 (5.37, 5.57)	-
Patient Race	White	4.80 (4.64, 4.96)	*Ref.*	3.96 (3.82, 4.10)	*Ref.*	2.33 (2.20, 2.46)	*Ref.*	5.60 (5.46, 5.75)	*Ref.*
	Black	4.55 (4.39, 4.70)	**.02**	3.65 (3.52, 3.79)	**.002**	2.15 (2.03, 2.27)	**.05**	5.35 (5.22, 5.49)	**.02**
Partner Race	White	4.67 (4.51, 4.82)	*Ref.*	3.77 (3.64, 3.91)	*Ref.*	2.27 (2.14, 2.39)	*Ref.*	5.49 (5.35, 5.64)	*Ref.*
	Black	4.68 (4.52, 4.83)	.94	3.84 (3.71, 3.98)	.47	2.21 (2.08, 2.33)	.50	5.46 (5.32, 5.61)	.76
Pt. Race X Partner Race	White X White	4.49 (4.27, 4.71)	*Ref.*	3.78 (3.59, 3.97)	*Ref.*	2.12 (1.95, 2.30)	*Ref.*	5.40 (5.20, 5.60)	*Ref.*
	White X Black	4.60 (4.39, 4.81)	.47	3.53 (3.35, 3.71)[b,c]	.07	2.18 (2.01, 2.35)	.67	5.31 (5.11, 5.50)[b]	.50
	Black X White	4.86 (4.64, 5.09)	**.02**	3.91 (3.71, 4.11)[b]	.36	2.29 (2.11, 2.47)	.19	5.52 (5.32, 5.73)	.41
	Black X Black	4.74 (4.51, 4.96)	.12	4.02 (3.82, 4.21)[c]	.09	2.36 (2.18, 2.54)	.07	5.68 (5.47, 5.89)[b]	.06

Table 2 Caption: This represents the adjusted means considering only the main effects of patient race and partner race. Separate models were constructed for each couple type (MSM, MSW, or WSM) and outcome (12 models in total). Models were adjusted for the following variables: respondents' year in training, sexual orientation, gender identity, and race. MSM = men who have sex with men; WSM = women who have sex with men; MSW = men who have sex with women.

a = difference is significant at $p < .05$; b = difference is significant at $p < .01$; c = difference is significant at $p < .001$.

Appendix 2 - Table 3. Moderation analyses (full analytic sample, *N* = 1,643).

	Anticipated Condomless Sex				Anticipated Extra-Relational Sex			
	Partial Effect (95%CI)	*p*	Conditional Effect (95%CI)	*p*	Partial Effect (95%CI)	*p*	Conditional Effect (95%CI)	*p*
**MSM**								
Pt Race (White)	-0.05 (-0.25, 0.15)	.61	-0.03 (-0.78, 0.71)	.93	-0.03 (-0.22, 0.16)	.78	0.36 (-0.36, 1.09)	.32
Partner Race	-0.02 (-0.22, 0.17)	.83	0.05 (-0.27, 0.38)	.74	-0.11 (-0.30, 0.08)	.26	0.02 (-0.30, 0.33)	.92
Pt. Race X Partner Race	-	-	-0.06 (-0.52, 0.40)	.81	-	-	-0.21 (-0.65, 0.23)	.35
Racism	0.04 (-0.18, 0.27)	.69	0.87 (-0.10, 1.85)	.08	0.13 (-0.08, 0.34)	.24	0.75 (-0.19, 1.70)	.12
Pt. Race X Racism	-	-	-1.05 (-2.46, 0.36)	.14	-	-	-1.01 (-2.37, 0.35)	.15
Partner Race X Racism	-	-	-0.65 (-1.26, -0.04)	**.04**	-	-	-0.34 (-0.93, 0.25)	.26
Pt. Race X Partner Race X Racism	-	-	0.90 (0.03, 1.77)	**.04**	-	-	0.51 (-0.34, 1.35)	.24
**MSW**								
Pt Race (White)	-0.09 (-0.30, 0.12)	.39	-0.21 (-1.01, 0.59)	.61	-0.14 (-0.33, 0.05)	.15	-0.17(-0.90, 0.56)	.65
Partner Race	-0.07 (-0.27, 0.14)	.54	-0.15 (-0.50, 0.20)	.42	0.13 (-0.06, 0.32)	.18	0.10 (-0.22, 0.42)	.54
Pt. Race X Partner Race	-	-	0.09 (-0.41, 0.59)	.73	-	-	0.03 (-0.43, 0.49)	.90
Racism	0.30 (0.06, 0.55)	**.02**	0.28 (-0.80, 1.36)	.61	-0.01 (-0.24, 0.21)	.91	0.13 (-0.85, 1.12)	.79
Pt. Race X Racism	-	-	-0.28 (-1.88, 1.32)	.73	-	-	-0.38 (-1.84, 1.08)	.61
Partner Race X Racism	-	-	0.03 (-0.66, 0.72)	.93	-	-	-0.08 (-0.71, 0.55)	.80
Pt. Race X Partner Race X Racism	-	-	0.15 (-0.84, 1.15)	.76	-	-	0.22 (-0.69, 1.13)	.64
**WSM**								
Pt Race (White)	-0.28 (-0.50, -0.05)	**.02**	-0.29 (-1.17, 0.59)	.52	-0.33 (-0.53, -0.13)	**.001**	0.09 (-0.69, 0.86)	.83
Partner Race	0.00 (-0.22, 0.22)	.99	0.06 (-0.32, 0.43)	.77	-0.08 (-0.27, 0.12)	.43	0.09 (-0.23, 0.42)	.57
Pt. Race X Partner Race	-	-	0.04 (-0.51, 0.59)	.89	-	-	-0.30 (-0.78, 0.18)	.22
Racism	0.23 (-0.03, 0.49)	.09	1.34 (0.19, 2.49)	**.02**	0.16 (-0.07, 0.38)	.18	0.02 (-1.00, 1.04)	.97
Pt. Race X Racism	-	-	-1.28 (-2.93, 0.38)	.13	-	-	0.38 (-1.08, 1.83)	.61
Partner Race X Racism	-	-	-0.70 (-1.44, 0.04)	.06	-	-	0.05 (-0.60, 0.70)	.88
Pt. Race X Partner Race X Racism	-	-	0.76 (-0.27, 1.80)	.15	-	-	-0.16 (-1.07, 0.75)	.73

Appendix 2 - Table 3. Moderation analyses (full analytic sample, *N* = 1,643), continued.

	Anticipated Non-Adherence				Assumed HIV Risk			
	Partial Effect (95%CI)	*p*	Conditional Effect (95%CI)	*p*	Partial Effect (95%CI)	*p*	Conditional Effect (95%CI)	*p*
	**MSM**							
Pt Race (White)	-0.04 (-0.21, 0.14)	.68	-0.20 (-0.88, 0.47)	.56	0.03 (-0.18, 0.24)	.77	0.33 (-0.45, 1.12)	.41
Partner Race	-0.19 (-0.37, -0.02)	**.03**	-0.26 (-0.55, 0.04)	.09	0.24 (0.03, 0.44)	**.02**	0.29 (-0.05, 0.63)	.10
Pt. Race X Partner Race	-	-	0.13 (-0.28, 0.55)	.53	-		-0.19 (-0.68, 0.29)	.43
Racism	0.10 (-0.10, 0.30)	.31	0.06 (-0.82, 0.94)	.90	0.19 (-0.05, 0.42)	.12	0.10 (-0.92, 1.13)	.84
Pt. Race X Racism	-	-	0.19 (-1.09, 1.46)	.77	-	-	-0.39 (-1.88, 1.09)	.61
Partner Race X Racism	-	-	0.08 (-0.47, 0.63)	.79	-	-	0.06 (-0.58, 0.70)	.85
Pt. Race X Partner Race X Racism	-	-	-0.22 (-1.00, 0.57)	.59	-	-	0.23 (-0.69, 1.15)	.62
	**MSW**							
Pt Race (White)	-0.17 (-0.34, 0.00)	**.05**	0.12 (-0.55, 0.79)	.73	0.14 (-0.06, 0.35)	.17	-0.08 (-0.88, 0.71)	.84
Partner Race	0.10 (-0.08, 0.27)	.27	0.24 (-0.05, 0.53)	.10	-0.08 (-0.29, 0.13)	.46	-0.03 (-0.38, 0.32)	.88
Pt. Race X Partner Race	-	-	-0.14 (-0.56, 0.28)	.52	-	-	0.06 (-0.43, 0.56)	.80
Racism	-0.04 (-0.25, 0.17)	.70	0.56 (-0.33, 1.45)	.22	0.04 (-0.21, 0.28)	.77	0.50 (-0.57, 1.58)	.36
Pt. Race X Racism	-	-	-0.36 (-1.72, 1.01)	.61	-	-	0.04 (-1.56, 1.64)	.96
Partner Race X Racism	-	-	-0.30 (-0.88, 0.27)	.30	-	-	-0.46 (-1.15, 0.23)	.19
Pt. Race X Partner Race X Racism	-	-	0.04 (-0.80, 0.88)	.92	-	-	0.28 (-0.72, 1.27)	.59
	**WSM**							
Pt Race (White)	-0.19 (-0.37, -0.01)	**.04**	-0.82 (-1.53, -0.10)	**.02**	-0.25 (-0.46, -0.05)	**.02**	-0.12 (-0.93, 0.70)	.78
Partner Race	0.06 (-0.12, 0.24)	.49	-0.07 (-0.38, 0.23)	.64	0.02 (-0.18, 0.23)	.82	0.18 (-0.17, 0.53)	.31
Pt. Race X Partner Race	-	-	0.36 (-0.08, 0.81)	.11	-	-	-0.15 (-0.66, 0.35)	.56
Racism	0.17 (-0.04, 0.38)	.11	-0.73 (-1.66, 0.20)	.12	0.02 (-0.22, 0.26)	.89	0.01 (-1.06, 1.08)	.98
Pt. Race X Racism	-	-	2.01 (0.68, 3.35)	**.003**	-	-	0.67 (-0.86, 2.20)	.39
Partner Race X Racism	-	-	0.54 (-0.06, 1.14)	.08	-	-	-0.09 (-0.78, 0.60)	.79
Pt. Race X Partner Race X Racism	-	-	-1.19 (-2.02, -0.35)	**.01**	-	-	-0.25 (-1.21, 0.71)	.61

Table 3 Caption: The coefficients for the moderation analyses which were conducted with the full analytic sample, including those who passed and those who failed the manipulation check item. A series of four moderation models were conducted (manuscript Figure 3) for each couple type (MSM, MSW, WSM) to evaluate the effects of patient and partner race as well as potential moderating effects of implicit racism on assumptions and assumed HIV risk. For all moderation analyses, White patient race was taken as the reference group. Racism indicates the implicit racism IAT *d*-score. All moderation models controlled for respondents' year in training, sexual orientation, gender identity, and race. MSM = men who have sex with men; WSM = women who have sex with men; MSW = men who have sex with women.
